# Thiosemicarbazones and selected tyrosine kinase inhibitors synergize in pediatric solid tumors: NDRG1 upregulation and impaired prosurvival signaling in neuroblastoma cells

**DOI:** 10.3389/fphar.2022.976955

**Published:** 2022-09-07

**Authors:** Maria Krchniakova, Silvia Paukovcekova, Petr Chlapek, Jakub Neradil, Jan Skoda, Renata Veselska

**Affiliations:** ^1^ Laboratory of Tumor Biology, Department of Experimental Biology, Faculty of Science, Masaryk University, Brno, Czechia; ^2^ International Clinical Research Center, St. Anne’s University Hospital, Brno, Czechia

**Keywords:** pediatric solid tumors, neuroblastoma, tyrosine kinase inhibitors, thiosemicarbazones, receptor tyrosine kinases, NDRG1

## Abstract

Tyrosine kinase inhibitors (TKIs) are frequently used in combined therapy to enhance treatment efficacy and overcome drug resistance. The present study analyzed the effects of three inhibitors, sunitinib, gefitinib, and lapatinib, combined with iron-chelating agents, di-2-pyridylketone-4,4-dimethyl-3-thiosemicarbazone (Dp44mT) or di-2-pyridylketone-4-cyclohexyl-4-methyl-3-thiosemicarbazone (DpC). Simultaneous administration of the drugs consistently resulted in synergistic and/or additive activities against the cell lines derived from the most frequent types of pediatric solid tumors. The results of a detailed analysis of cell signaling in the neuroblastoma cell lines revealed that TKIs inhibited the phosphorylation of the corresponding receptor tyrosine kinases, and thiosemicarbazones downregulated the expression of epidermal growth factor receptor, platelet-derived growth factor receptor, and insulin-like growth factor-1 receptor, leading to a strong induction of apoptosis. Marked upregulation of the metastasis suppressor N-myc downstream regulated gene-1 (NDRG1), which is known to be activated and upregulated by thiosemicarbazones in adult cancers, was also detected in thiosemicarbazone-treated neuroblastoma cells. Importantly, these effects were more pronounced in the cells treated with drug combinations, especially with the combinations of lapatinib with thiosemicarbazones. Therefore, these results provide a rationale for novel strategies combining iron-chelating agents with TKIs in therapy of pediatric solid tumors.

## 1 Introduction

The overall 5-year survival rate of childhood cancer patients has considerably increased over the past decades, reaching approximately 85% (Miller et al., 2019). Despite improved outcomes, the development of therapy resistance remains a major obstacle for a wide variety of pediatric solid tumors, including neuroblastoma (Alisi et al., 2013), medulloblastoma (Othman et al., 2014), osteosarcoma (Brambilla et al., 2012), and rhabdomyosarcoma (Gallego et al., 2004). The management of pediatric cancers is aimed at eradicating the tumor and must also carefully consider organ toxicity and the development of chronic health conditions in young individuals (Oeffinger et al., 2007; Miller et al., 2019). Therefore, personalized medicine and drug combination strategies are being investigated to improve the efficacy of therapy while reducing short- and/or long-term adverse effects in patients.

Tyrosine kinase inhibitors (TKIs) are a group of targeted therapeutics that attenuate the aberrant activity of tyrosine kinases in cancer; thus, TKIs are a promising approach in pediatric oncology (Yamaoka et al., 2018). We have previously identified receptor tyrosine kinases (RTKs) as druggable targets in relapsed or refractory childhood solid tumors (Mudry et al., 2017; Neradil et al., 2019). However, the use of TKIs as monotherapy is notoriously associated with the development of multidrug resistance (MDR) in patients (Jiao et al., 2018). Various mechanisms underlie MDR, including enhanced drug elimination by upregulated ABC transporters, which efflux the drugs out of the cells (Wu et al., 2011), or by lysosomal sequestration (Zhitomirsky and Assaraf, 2016). Particular physico-chemical properties of some drugs may be responsible for sequestration into the lysosomes where the drugs become charged and thus trapped, preventing the drugs from reaching their targets (Zhitomirsky and Assaraf, 2016). On the other hand, a combination of TKIs with standard chemotherapeutics has been shown to improve the clinical response, resulting in more efficient treatment of the tumors prevalent in adults (Krchniakova et al., 2020). Furthermore, new compounds with proven anticancer effects are emerging as the candidates to be tested in these combination therapies (Tong et al., 2017), and these new compounds include thiosemicarbazones (Paukovcekova et al., 2020).

Thiosemicarbazone iron chelators of the DpT class, including di-2-pyridylketone-4,4-dimethyl-3-thiosemicarbazone (Dp44mT) and its analog di-2-pyridylketone-4-cyclohexyl-4-methyl-3-thiosemicarbazone (DpC), have been shown to be effective and selective against a wide variety of tumors both *in vitro* and *in vivo* (Yu et al., 2009; Liu et al., 2012; Dixon et al., 2013; Potuckova et al., 2014; Jansson et al., 2015; Guo et al., 2016; Xu et al., 2018; Paukovcekova et al., 2020). In addition to chelation of iron and copper, which are critical for tumor cell proliferation (Lovejoy et al., 2011; Lane et al., 2015), these agents have been shown to potently induce the expression of the metastasis suppressor N-myc downstream regulated gene-1 (NDRG1) (Bae et al., 2013; Park et al., 2018) and to suppress the key oncogenic signaling pathways (Chen et al., 2012; Dixon et al., 2013; Liu et al., 2015; Kovacevic et al., 2016; Menezes et al., 2017, 2019b; Chekmarev et al., 2021; Geleta et al., 2021). Multiple reports have demonstrated that Dp44mT and DpC potentiate the effects of anticancer drugs both *in vitro* and *in vivo* (Lovejoy et al., 2012; Potuckova et al., 2014; Seebacher N. A. et al., 2016; Maqbool et al., 2020; Paukovcekova et al., 2020), and our previous study showed a promising synergy between thiosemicarbazones and celecoxib in pediatric cancer cells (Paukovcekova et al., 2020).

Both Dp44mT and DpC were suggested to accumulate in the lysosomes, where they form redox-active complexes with copper, which lead to the generation of reactive oxygen species (ROS) that permeabilize the lysosomal membrane and subsequently induce apoptosis (Yamagishi et al., 2013; Jansson et al., 2015). Hence, Dp44mT was shown to restore the sensitivity of carcinoma cells to doxorubicin that is otherwise trapped in the lysosomes (Jansson et al., 2015; Seebacher N. A. et al., 2016). Interestingly, lysosomal trapping was also shown to mediate resistance to several TKIs, including sunitinib (SUN), gefitinib (GEF), and lapatinib (LAP) (Gotink et al., 2011; Kazmi et al., 2013).

Based on these published findings, we decided to examine the potential anticancer interactions of Dp44mT and DpC with the three TKIs already in use in pediatric oncology: 1) GEF targeting epidermal growth factor receptor (EGFR) (Pollack et al., 2010), 2) LAP that targets EGFR and ErbB2 (Fouladi et al., 2013), and 3) a multikinase inhibitor SUN that primarily inhibits platelet-derived growth factor receptors (PDGFRs), vascular endothelial growth factor receptors (VEGFRs), c-Kit, or FLT3 (Mudry et al., 2017; Verschuur et al., 2019). The results of the tests of multiple combination strategies in the cell lines derived from pediatric solid tumors performed in the present study demonstrated that Dp44mT and DpC significantly potentiated the activity of selected TKIs. The results of the present study also identified several targets that are synergistically affected by these drugs. These findings provide promising evidence for novel treatment strategies that combine TKIs with iron-chelating agents, such as Dp44mT or DpC, to treat pediatric solid tumors.

## 2 Materials and methods

### 2.1 Cell lines and cell culture

Five cancer cell lines derived from pediatric solid tumors were used in the present study. The neuroblastoma SH-SY5Y (ECACC 94030304), SK-N-BE(2) (ECACC 95011815), and rhabdomyosarcoma RD (ECACC 85111502) cell lines were purchased from the European Collection of Authenticated Cell Cultures (ECACC, Salisbury, United Kingdom). The medulloblastoma DAOY (ATCC HTB-186^™^) and osteosarcoma Saos-2 (ATCC HTB-85^™^) cell lines were obtained from the American Type Culture Collection (ATCC, Manassas, VA, United States). All cell lines were authenticated by STR profiling and routinely tested negative for mycoplasma contamination by PCR.

All reagents for cell culture were purchased from Biosera (Nuaille, France). DAOY and Saos-2 cells were cultured in Dulbecco’s modified Eagle’s medium (DMEM; low glucose, cat. no. LM-D1100) supplemented with 10% fetal calf serum (FCS; cat. no. FB-1101); RD cells were maintained in DMEM (high glucose, cat. no. LM-D1112) with 10% FCS, and SH-SY5Y and SK-N-BE(2) cells were cultured in a mixture of DMEM/F12 (1:1, cat no. LM-D1224) supplemented with 20% FCS. All media were further supplemented with 2 mM glutamine (cat. no. XC-T1715), penicillin (100 IU/ml), and streptomycin (100 μg/ml; cat. no. XC-A4122). The media used for DAOY, RD, SH-SY5Y, and SK-N-BE(2) cells also contained 1% nonessential amino acids (cat. no. XC-E1154). The cells were maintained under standard cell culture conditions at 37°C in a humidified atmosphere containing 5% CO_2_ and were subcultured 1–2 times weekly.

### 2.2 Chemicals

The tyrosine kinase inhibitors SUN (cat. no. 12328), GEF (cat. no. 4765), and LAP (cat. no. 12121) were purchased from Cell Signaling Technology (Danvers, MA, United States). Thiosemicarbazones Dp44mT (cat. no. SML0186) and DpC (cat. no. SML0483) and Valspodar (VAL; cat. no. SML0572) were purchased from Sigma–Aldrich (St. Louis, MO, United States). All reagents were prepared as stock solutions in dimethyl sulfoxide (DMSO; purchased from Sigma–Aldrich) at the concentrations of 10 mM (LAP), 75 mM (SUN) or 100 mM (GEF, Dp44mT, DpC, and VAL).

### 2.3 Cell proliferation assays

Cell proliferation was evaluated after drug treatment using the 3-[4,5-dimethylthiazol-2-yl]-2,5-diphenyltetrazolium bromide (MTT) assays. The cells were seeded at variable densities to ensure that they remained in the log growth phase during the drug treatments. For 24-hour treatment, the cells were seeded in 96-well plates at a density of 2×10^4^ cells/well (SH-SY5Y, SK-N-BE(2), Saos-2, and RD cells) or 5×10^3^ cells/well (DAOY cells). For 72-hour treatment, the cells were seeded at a density of 5×10^3^ cells/well (SH-SY5Y, SK-N-BE(2), Saos-2, and RD cells) or 8×10^2^ cells/well (DAOY cells). After incubation with the drugs, the cells were incubated with MTT (0.5 mg/ml; cat. no. M2128, purchased from Sigma–Aldrich) for 3 h under standard cell culture conditions. Subsequently, the medium was removed, and formazan crystals were dissolved in 200 µl of DMSO. The absorbance was measured at 570 nm, and the reference absorbance was measured at 620 nm using a Sunrise absorbance reader (Tecan, Männedorf, Switzerland).

### 2.4 IC_50_ determination

The MTT assay was used to determine the IC_50_ values (concentration at which the cellular population was reduced by 50%) for each drug (SUN, GEF, LAP, Dp44mT, or DpC). 24 h after seeding, the medium was replaced with 200 µl of the fresh medium containing appropriate concentrations of the drugs alone. After incubation for 72 and/or 24 h under standard cell culture conditions, the cells were incubated with MTT and analyzed as described above. The IC_50_ value was assessed using CalcuSyn software (version 2.0, Biosoft, Cambridge, United Kingdom).

### 2.5 Combined treatment protocols

**FIGURE 1:**
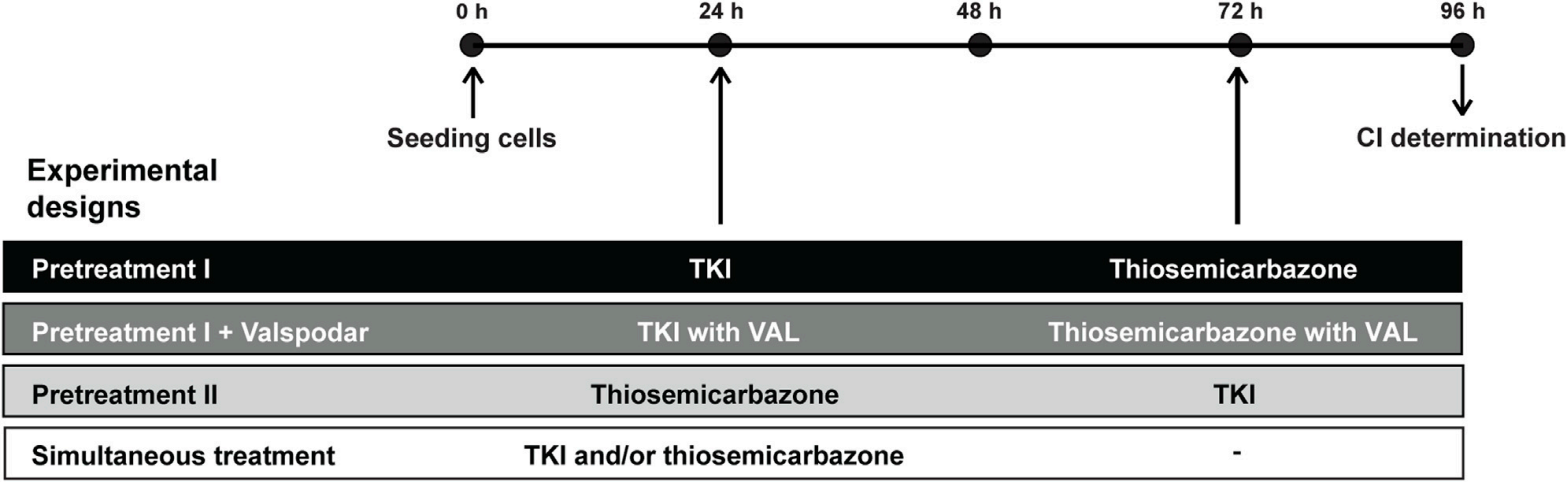
Experimental designs and timeline of combined treatments used to evaluate the interactions between thiosemicarbazones (Dp44mT and DpC) and TKIs (SUN, GEF, and LAP). According to sequential treatment designs with various pretreatments, the cells were seeded, and the following compounds were added: a TKI (Pretreatment I), a TKI with 0.2 Valspodar (VAL; Pretreatment I + VAL), or a thiosemicarbazone (Pretreatment II). 48 h later, another drug was added to the assay: a thiosemicarbazone (Pretreatment I), a thiosemicarbazone with 0.2 VAL (Pretreatment I + VAL), or a TKI (Pretreatment II). In the case of Simultaneous treatment, both a thiosemicarbazone and a TKI were added together. At the end of the tests, the drug interactions were assessed as a combination index (CI). Drug concentrations used to determine CIs in the individual treatment designs were derived from the respective IC_50_ values listed in Table 2 (for detailed methodology, see Materials and Methods Section 2.5 and Section 2.6).

The MTT assay was used to quantify the synergy between thiosemicarbazones and TKIs. Based on the initially calculated IC_50_ values of each drug, the drug concentrations used in the combined treatment experiments corresponded to 1/8-, ¼-, ½-, 1-, 2-, 4-, and 8-fold of the IC_50_ using methodology as reported previously (Paukovcekova et al., 2020). The cells were seeded as described above. After 24 h, the medium was replaced, and the cells were incubated with the drugs alone or in combination under standard cell culture conditions. Different experimental designs were utilized to assess the effects of the combinations of thiosemicarbazones Dp44mT and DpC with TKIs SUN, GEF, and LAP (Figure 1).

In the experiments with sequential administration of the drugs (Figure 1, Pretreatment designs), the cells were initially treated with an appropriate concentration of SUN, GEF, or LAP (Pretreatment I), SUN, GEF, or LAP with 0.2 µM VAL (Pretreatment I + VAL), or Dp44mT or DpC (Pretreatment II). After 48 h, 100 µl of the fresh medium was added, and the medium contained an appropriate concentration of Dp44mT or DpC (Pretreatment I), Dp44mT or DpC with 0.2 µM VAL (Pretreatment I + VAL), or SUN, GEF, or LAP (Pretreatment II). In the simultaneous treatment experiments (Figure 1, Simultaneous treatment), the cells were treated with an appropriate concentration of individual drugs or their combinations and incubated for another 72 h. After the drugs were incubated according to the corresponding experimental design, cell proliferation was analyzed by the MTT assays as described above.

To investigate the molecular effects of TKIs in combination with thiosemicarbazones, the cells were seeded in Petri dishes (90 mm in diameter) and allowed to adhere overnight. To reproduce the simultaneous treatment design (Figure 1), thiosemicarbazones and TKIs alone or in combination were added at the corresponding IC_50_ concentrations. The cells were incubated under standard cell culture conditions in the presence or in the absence (control) of the indicated drugs for 72 h before being processed for immunoblotting.

### 2.6 Calculation of combination index

CalcuSyn software (version 2.0, Biosoft, Cambridge, United Kingdom) and the Chou Talalay method were used to calculate the combination index (CI) values as described previously (Paukovcekova et al., 2020). A 1:1 ratio of the drugs was used for combination treatments, and the CI values were calculated based on the growth inhibition curves. The dose-effect relationship for each drug alone was compared to the corresponding combination to identify the synergistic (CI < 0.9), additive (CI: 0.9–1.1), or antagonistic (CI > 1.1) interactions between thiosemicarbazones and TKIs (Chou, 2006).

### 2.7 Phospho-RTK arrays

The relative levels of phosphorylation of 49 RTKs (Supplementary Figure S1) were assayed using a Proteome Profiler™ human phospho-RTK array kit (cat. no. ARY001B) purchased from R&D Systems (Minneapolis, MN, United States). Treated and/or untreated control cells were lysed using lysis buffer 17 and processed according to the manufacturer’s instructions. Each array was incubated with 300 µg of the whole-cell lysate. The relative levels of RTK phosphorylation were quantified using Fiji software (Schindelin et al., 2012), and analysis was performed as described previously (Neradil et al., 2019).

### 2.8 Western blotting and immunodetection

Whole-cell lysates of treated and untreated control cells were loaded on 10% polyacrylamide gels (10–20 µg/well), electrophoresed, and blotted on the polyvinylidene difluoride membranes (purchased from Bio–Rad Laboratories, Munich, Germany). Depending on the primary antibody, the membranes were blocked either with 5% nonfat dry milk or with bovine serum albumin (BSA; Sigma–Aldrich) in phosphate-buffered saline (PBS) containing 0.1% Tween-20 (Sigma–Aldrich) for 1 h at room temperature and then incubated at 4°C overnight with the corresponding primary antibodies listed in Table 1. Then, the membranes were incubated with the corresponding secondary antibodies (Table 1) for 1 h at room temperature. Chemiluminescence detection was performed using Amersham™ ECL™ Prime Western blotting detection reagent (purchased from GE Healthcare, Little Chalfont, United Kingdom) according to the manufacturer’s instructions. Densitometry analyses were performed using Fiji software (Schindelin et al., 2012), and the densities of protein bands of interest were normalized to that of the loading control. Glyceraldehyde-3-phosphate dehydrogenase (GAPDH) and alpha tubulin were used as the loading controls. Biological replicates were normalized using the sum of all data points in a replicate as described by Degasperi et al. (2014).

**TABLE 1:** Primary and secondary antibodies used in the experiments. All antibodies, except an anti-alpha tubulin antibody (Abcam, Cambridge, MA, United States), were purchased from Cell Signaling Technologies (Danvers, MA, United States). BSA, bovine serum albumin; HRP, horseradish peroxidase; Mo, mouse; Mono, monoclonal; NFDM, nonfat dry milk; Poly, polyclonal; Rb, rabbit.

Primary antibodies					
Antigen	Type/Host	Clone	Cat. No.	Dilution	Blocking
AKT (pan)	Mono/Rb	C67E7	4691	1:1000	NFDM
Phospho-AKT (Ser473)	Mono/Rb	D9E	4060	1:1000	BSA
Alpha tubulin	Mono/Mo	DM1A	ab7291	1:10,000	NFDM
Cleaved Caspase 3 (Asp175)	Mono/Rb	5A1E	9664	1:1000	NFDM
EGFR	Mono/Rb	C74B9	2646	1:1000	NFDM
Phospho-EGFR (Tyr1068)	Mono/Mo	1H12	2236	1:1000	BSA
Phospho-EGFR (Tyr1148)	Poly/Rb	—	4404	1:1000	BSA
ERK1/2	Mono/Rb	137F5	4695	1:1000	NFDM
Phospho-ERK1/2 (Thr202/204)	Mono/Rb	D13.14.4E	4370	1:1000	BSA
GAPDH	Mono/Rb	14C10	2118	1:5000	NFDM
IGF-1Rβ	Mono/Rb	D23H3	9750	1:1000	NFDM
MEK1/2	Poly/Rb	—	9122	1:1000	NFDM
Phospho-MEK1/2 (Ser217/221)	Poly/Rb	—	9121	1:1000	BSA
NDRG1	Mono/Rb	D8G9	9485	1:1000	NFDM
Phospho-NDRG1 (Thr346)	Mono/Rb	D98G11	5482	1:1000	BSA
PDGFRβ	Mono/Rb	28E1	3168	1:1000	NFDM
Phospho-PDGFRβ (Tyr751)	Mono/Rb	C63G6	4549	1:1000	NFDM

Secondary Antibodies					
Specificity	Host	Conjugate	Cat. No.	Dilution	Blocking
Anti-Mo IgG	Horse	HRP	7076	1:5000	NFDM
Anti-Rb IgG	Goat	HRP	7074	1:5000	NFDM

### 2.9 Statistical analysis

Quantitative data are shown as the mean ± standard deviation (SD) of three independent experiments. The data of the MTT assays of the combination treatments and the results of densitometry were analyzed using one-way ANOVA followed by Dunnett’s post-hoc test. All statistical analyses were performed using GraphPad Prism 8.0.2 software (GraphPad Software Inc., San Diego, CA, United States), and *p* < 0.05 was considered statistically significant.

## 3 Results

### 3.1 Active prosurvival RTK signaling and sensitivity to both thiosemicarbazones and TKIs is detected in pediatric solid tumor cells

**FIGURE 2:**
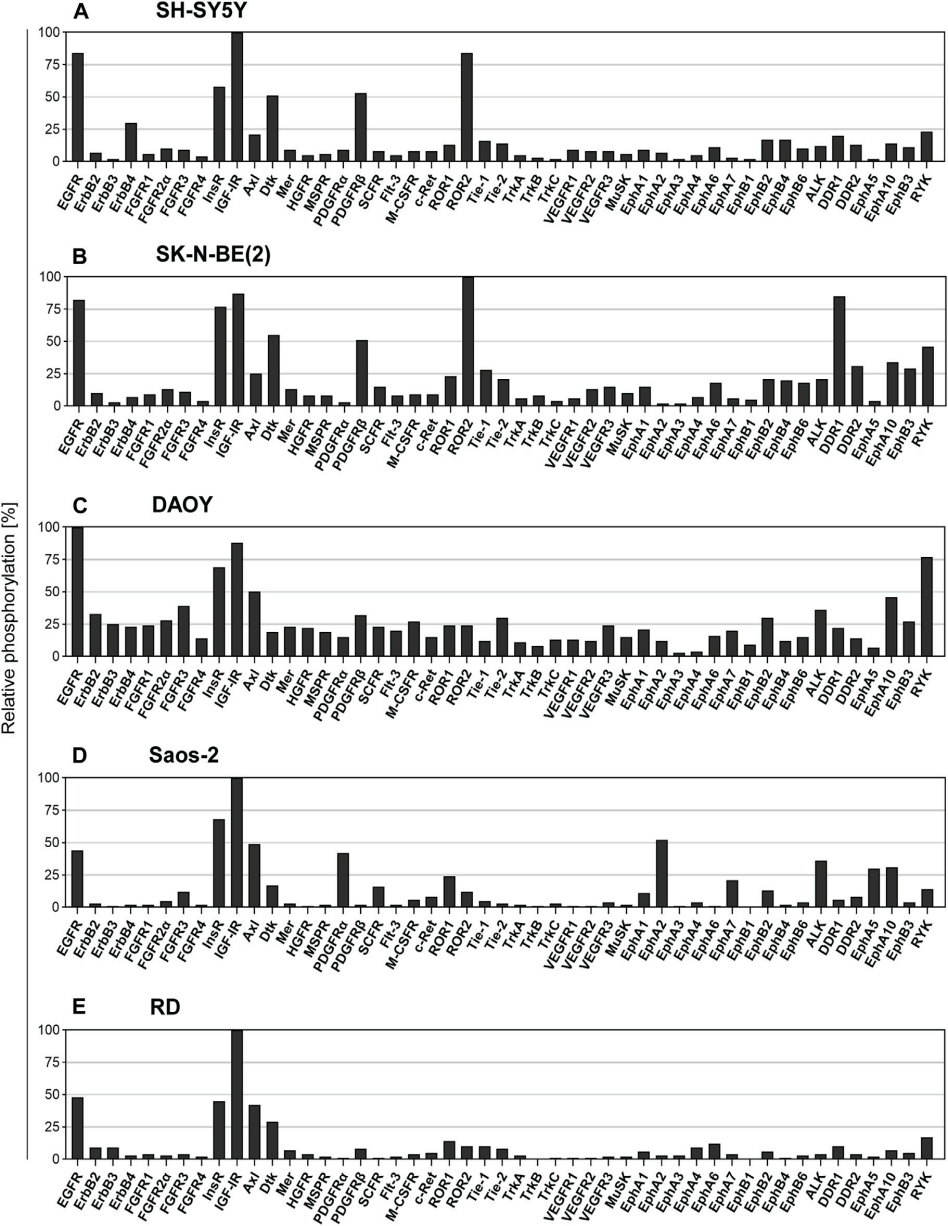
Screening of the levels of the RTK phosphorylation in untreated cell lines. The relative phosphorylation levels of 49 RTKs were assayed in the cell lines: **(A)** SH-SY5Y and **(B)** SK-N-BE (2) cells derived from neuroblastoma, **(C)** DAOY cells derived from medulloblastoma, **(D)** Saos-2 cells derived from osteosarcoma, and **(E)** RD cells derived from rhabdomyosarcoma. Columns represent the levels of relative phosphorylation of RTKs assessed as described in Materials and Methods. The corresponding array images are shown in Supplementary Figure S2.

The drugs investigated in the present study, including the TKIs SUN, GEF, and LAP and thiosemicarbazones Dp44mT and DpC, have been demonstrated to affect the critical oncogenic signaling pathways (Pollack et al., 2010; Chen et al., 2012; Dixon et al., 2013; Fouladi et al., 2013; Lui et al., 2015b; Kovacevic et al., 2016; Menezes et al., 2017; Mudry et al., 2017; Menezes et al., 2019a; Verschuur et al., 2019; Geleta et al., 2021). Therefore, we initially assayed the basal activity of 49 key RTKs in untreated cell lines derived from pediatric solid tumors, including the SH-SY5Y and SK-N-BE(2) neuroblastoma, Saos-2 osteosarcoma, RD rhabdomyosarcoma, and DAOY medulloblastoma cell lines (Figure 2; Supplementary Figure S2).

Overall, the results of screening using the human phosphoprotein arrays revealed high phosphorylation of multiple pro-oncogenic RTKs, several of which are known to be attenuated by the drugs used in the present study. The ErbB family of RTKs are known to be targeted by GEF, LAP, and both thiosemicarbazones (Pollack et al., 2010; Fouladi et al., 2013; Kovacevic et al., 2016; Menezes et al., 2017), and EGFR was consistently identified as one of the five most phosphorylated RTKs in all tested cell lines (Figure 2). In contrast, the activation of other ErbB family members was substantially lower (Figure 2). Regarding the targets of SUN (Mudry et al., 2017; Verschuur et al., 2019), we detected relatively active PDGFRα in Saos-2 cells (Figure 2D) and PDGFRβ in both SH-SY5Y and SK-N-BE(2) neuroblastoma cells (Figures 2A,B); however, the phosphorylation of other RTKs targeted by SUN, e.g., VEGFRs or FLT3, was minimal (Figure 2). Notably, IGF-1R signaling is modulated by thiosemicarbazones (Geleta et al., 2021). The levels of phosphorylation of IGF-1R, and insulin receptor (InsR) were very high in all cell types (Figure 2). These results indicated that the activation of prosurvival RTK signaling was shared among all pediatric tumor cell lines, further supporting the rationale for the investigation of the interactions of selected RTK-targeting drugs in the present study.

Then, we determined the antiproliferative effect of thiosemicarbazones and selected TKIs used as single treatment agents and calculated the concentrations of the drugs at which cell proliferation was reduced by 50% (IC_50_). To determine the IC_50_ values needed for the subsequent combined treatment experiments, cell proliferation was evaluated after 24- and/or 72-hour incubation with individual drugs. In the case of TKI treatment, all tested cells were most sensitive to SUN and least sensitive to GEF after incubation for both 24 and 72 h (Table 2). Dp44mT and DpC administered for 24 h were most effective in DAOY medulloblastoma cells, whereas other cell lines showed ∼6- to 170-fold lower sensitivity (Table 2). After 72 h of incubation, SH-SY5Y and SK-N-BE(2) neuroblastoma cells showed higher sensitivity to thiosemicarbazones than other cell types (Table 2). As expected, all examined drugs demonstrated higher efficacy (corresponding to lower IC_50_ values) after prolonged incubation. This phenomenon was particularly evident in the case of thiosemicarbazones; the corresponding IC_50_ values for these compounds shifted from a micromolar range after 24-hour treatments to a nanomolar range after 72-hour treatments (Table 2). Furthermore, after incubation for 72 h, both Dp44mT and DpC were significantly more effective than SUN, GEF, or LAP in all cell lines (Table 2).

**TABLE 2:** The IC_50_ values. The concentrations of the drugs corresponding to a reduction in cell proliferation by 50%. The IC_50_ values were determined for each cell line after incubation with TKIs (SUN, GEF, and LAP) and thiosemicarbazones (Dp44mT and DpC) alone for 72 and/or 24 h at 37°C. Bullets indicate drug concentrations used in different combined treatment designs in accordance with Figure 1 (black: Pretreatment I; dark grey: Pretreatment + Valspodar; light grey: Pretreatment II; white: Simultaneous treatment).

Drugs	Time (h)	IC_50_				
		SH-SY5Y	SK-N-BE(2)	Daoy	Saos-2	RD
SUN	24 h	8.1 µM●	9.8 µM●	—	—	—
	72 h	4.5 µM● ● ○	5.6 µM● ● ○	3.7 µM● ● ○	2.0 µM● ● ○	2.4 µM● ● ○
GEF	24 h	41.7 µM●	86.7 µM●	—	—	—
	72 h	15.8 µM● ● ○	20.0 µM● ● ○	14.0 µM● ● ○	15.2 µM● ● ○	15.5 µM● ● ○
LAP	24 h	36.9 µM●	27.2 µM●	—	—	—
	72 h	9.7 µM● ● ○	8.3 µM● ● ○	10.0 µM● ● ○	11.9 µM● ● ○	8.7 µM● ● ○
Dp44mT	24 h	28.6 µM● ●	47.6 µM● ●	0.6 µM● ●	101.8 µM● ●	105.7 µM● ●
	72 h	1.1 nM● ○	2.3 nM● ○	11.1 nM○	15.3 nM○	7.2 nM○
DpC	24 h	10.7 µM● ●	31.0 µM● ●	1.6 µM● ●	30.8 µM● ●	24.9 µM● ●
	72 h	8.6 nM● ○	6.3 nM● ○	14.6 nM○	21.5 nM○	13.1 nM○
Treatment designs in which the respective concentrations were used:						
● Pretreatment I● Pretreatment II						
● Pretreatment I + Valspodar○ Simultaneous treatment						

**FIGURE 3:**
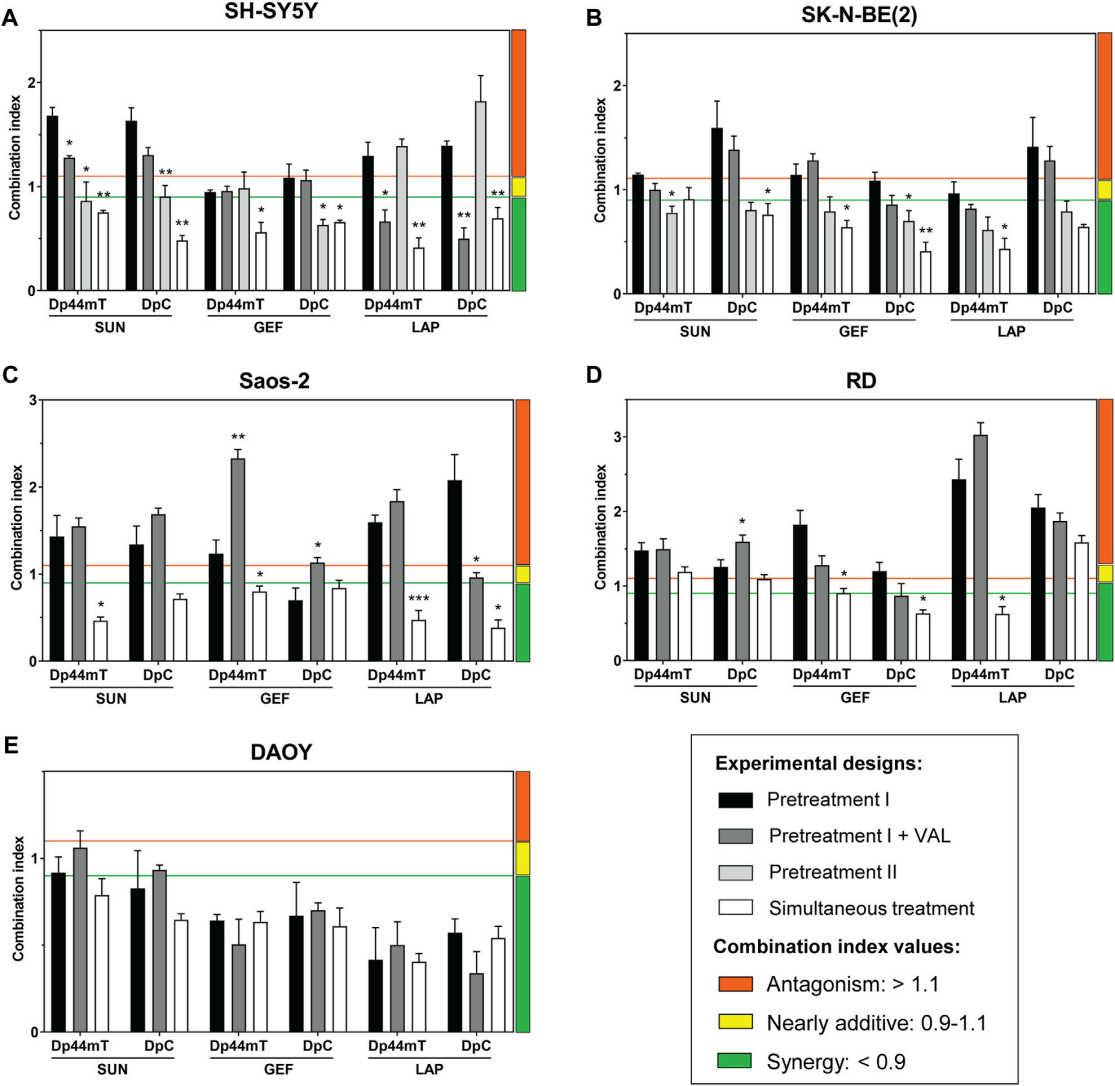
Combined treatments using thiosemicarbazones (Dp44mT and DpC) and TKIs (SUN, GEF, and LAP) of the cells derived from pediatric solid tumors. Combined treatments were evaluated in following cell lines **(A)** SH-SY5Y, **(B)** SK-N-BE(2), **(C)** Saos-2, **(D)** RD, and **(E)** DAOY. The drug combinations were applied using **(**i**)** sequential (Pretreatment I, Pretreatment I + Valspodar (VAL), or Pretreatment II) or (ii) simultaneous experimental designs (see Materials and Methods). IC_50_ values that served to determine drug concentrations used in the respective combined treatment designs are listed in Table 2. The interactions between the corresponding drugs were evaluated as a combination index (CI) and were calculated based on the growth inhibition curves. A 1:1 ratio of the drugs and the Chou-Talalay method were used to identify the synergistic (green), nearly additive (yellow), or antagonistic activity (orange) using CalcuSyn Software. Columns represent the CI values ±SD (*n* = 3). The data were analyzed using one-way ANOVA followed by Dunnett’s post-hoc test. *p < 0.05, **p < 0.01 and ***p < 0.0001 indicate significant differences compared to the results obtained according to the Pretreatment I experimental design.

Based on these data, we comprehensively evaluated the interactions between thiosemicarbazones and TKIs using several combined treatment designs (Figure 1): 1) sequential treatment protocols (Pretreatment I, Pretreatment I + VAL, and Pretreatment II) and 2) a simultaneous treatment protocol (Simultaneous treatment). The results of these experiments were recalculated as the CIs under various settings to assess the effects of the combinations of Dp44mT or DpC thiosemicarbazones with selected TKIs (Figure 3; Supplementary Table S1).

### 3.2 Variable effects of sequential treatments with thiosemicarbazones and TKIs on pediatric solid tumor cells

Based on the studies that have demonstrated that drug resistance to TKIs is mediated by lysosomal sequestration (Krchniakova et al., 2020) and on the ability of thiosemicarbazones to release the drugs, such as doxorubicin, sequestered into lysosomes (Jansson et al., 2015; Seebacher N. et al., 2016, Seebacher et al., 2016 N. A.), we hypothesized that the Pretreatment I strategy (Figure 1, Supplementary Table S1) may be a potent approach to enhance the anticancer effects of TKIs.

To test this hypothesis, the cells were initially treated with individual TKIs for 48 h and subsequently treated with Dp44mT or DpC for the next 24 h (Figure 1). However, the results of CI analyses revealed that this sequential treatment induced predominantly antagonistic drug interactions in all tested cell lines with the exception of DAOY medulloblastoma cells (Figure 3). These results contradicted our initial hypothesis.

SH-SY5Y cells express P-glycoprotein (Pgp) (Dalzell et al., 2015), which is an extensively studied ABC transporter involved in drug resistance (Wu et al., 2011). In addition to the drug efflux activity of Pgp at the plasma membrane, Pgp at the lysosomal membranes has been implicated in trapping of TKIs (Krchniakova et al., 2020) and inducing the accumulation of thiosemicarbazones in the lysosomes (Jansson et al., 2015; Seebacher N. A. et al., 2016, Seebacher et al., 2016 N.). To determine whether Pgp activity plays a role in the effects of the tested drugs, the selective Pgp inhibitor VAL at a concentration of 0.2 µM (Jansson et al., 2015) was added to the assay (Pretreatment I + VAL design; Figure 1). The most pronounced differences between the VAL(−) and VAL(+) conditions were detected in case of LAP + Dp44mT or DpC combinations in Pgp-expressing SH-SY5Y cells, where moderate antagonistic interactions changed to synergy (Figure 3A). In other cell lines, no consistent trends in the changes in the CI values were detected in case of the VAL(−) and VAL(+) treatment strategies (Figure 3).

To further elaborate on these results, we compared effective concentrations of the tested drugs under VAL(−) and VAL(+) conditions in Pgp-expressing SH-SY5Y cells. Unlike SUN and GEF, which are able to inhibit Pgp (Kitazaki et al., 2005; Shukla et al., 2009), thiosemicarbazones are transported by Pgp (Jansson et al., 2015; Seebacher N. A. et al., 2016), and LAP has been described as both Pgp substrate and inhibitor (Dai et al., 2008; Radic-Sarikas et al., 2017). Despite reported differences in the interactions of these drugs with Pgp, no significant changes in the efficacy of the drugs were detected by comparison of VAL-treated and control SH-SY5Y cells (Supplementary Figure S3). However, Pgp is known to export a wide variety of xenobiotics, metabolites, and toxins (Aller et al., 2009), and blockade of these functions by VAL could have explained enhanced cytotoxic effects of combined treatments in SH-SY5Y cells with inhibited Pgp (Pretreatment I + VAL design; Figure 3A). The inhibition of Pgp has been shown to prevent the accumulation of Dp44mT or DpC in the lysosomes, leading to a significant reduction in the cytotoxicity of these drugs in carcinoma cells (Jansson et al., 2015); however, the results of the present study suggested that this Pgp-dependent mechanism of action was not present in pediatric tumor cells. The inhibition of Pgp did not reduce the sensitivity of the tested cells to combined treatments with thiosemicarbazones. In the case of Pgp-overexpressing SH-SY5Y cells, the blockade of the Pgp activity even potentiated the effects of the drug combinations (Figure 3). These findings in combination with the failure of thiosemicarbazones to improve the efficacy of TKIs in TKI-pretreated cells indicated that the anticancer activity of thiosemicarbazones in pediatric solid tumor cells is most likely not facilitated through a lysosomal burst.

Since the treatments with thiosemicarbazones following the TKI pretreatment (Pretreatment I design) did not result in synergistic interactions in most cell lines, we explored another sequential design, Pretreatment II (Figure 1; Supplementary Table S1). According to this strategy, the drugs were administrated sequentially in reverse order: the treatment with Dp44mT or DpC for 48 h was followed by the addition of a TKI for the next 24 h (Figure 1). Considering that the drugs used in the present study interact with Pgp (Seebacher N. A. et al., 2016; Krchniakova et al., 2020), the neuroblastoma cell lines with different levels of Pgp expression, including SH-SY5Y (high Pgp expression) and SK-N-BE(2) cells (low Pgp expression) (Bates et al., 1989; Dalzell et al., 2015), were selected for these analyses.

Interestingly, in SK-N-BE(2) cells, the treatment according to the Pretreatment II design induced a significant decrease in the CI values compared to Pretreatment I, resulting in the uniform synergistic interactions between the tested drug combinations (Figure 3B). Additive/synergistic effects were also observed in SH-SY5Y cells when SUN or GEF was combined with Dp44mT or DpC (Figure 3A). These results suggested that thiosemicarbazones apparently sensitized pediatric solid tumor cells to TKIs. However, the combinations of LAP and thiosemicarbazones remained antagonistic in SH-SY5Y cells (Figure 3A), further emphasizing the role of Pgp in drug resistance.

Overall, sequential treatment protocols induced variable and generally unsatisfactory interactions between thiosemicarbazones and TKIs across the pediatric cancer cell lines, thus excluding the therapeutic potential of this approach.

### 3.3 Simultaneous administration of thiosemicarbazones and TKIs induces evident synergistic effects in pediatric solid tumor cells and apoptosis in neuroblastoma cells

Our previous report demonstrated that Dp44mT and DpC added simultaneously with celecoxib produce synergistic effects on pediatric cancer cells (Paukovcekova et al., 2020). Therefore, we implemented a similar strategy to examine the interactions between thiosemicarbazones and TKIs in all tested cell lines. According to this Simultaneous treatment design, the drugs were added to the assay at the same time, and the cells were incubated for 72 h (Figure 1, Supplementary Table S1).

imultaneous treatment was the most effective combination strategy in all cell lines tested in the present study. The effects of thiosemicarbazones and TKIs on DAOY cells were uniformly synergistic, with minimal differences versus the effects observed in the experiments performed according to the Pretreatment I design protocol (Figure 3E). Importantly, a significant difference between the two strategies was observed in SH-SY5Y (Figure 3A), SK-N-BE(2) (Figure 3B), and Saos-2 cells (Figure 3C), resulting in the uniformly synergistic and/or additive interactions in these cells in the experiments performed according to the Simultaneous treatment protocol. Furthermore, the synergy was achieved even after combining Dp44mT or DpC with LAP in SH-SY5Y cells; this drug combination produced antagonistic effects in the experiments performed according to the sequential treatment protocols (Figure 3A). The effects on RD cells were not as uniform as the effects on other cell lines; however, this Simultaneous approach produced a decrease in the CI values in most combinations (Figure 3D).

**FIGURE 4:**
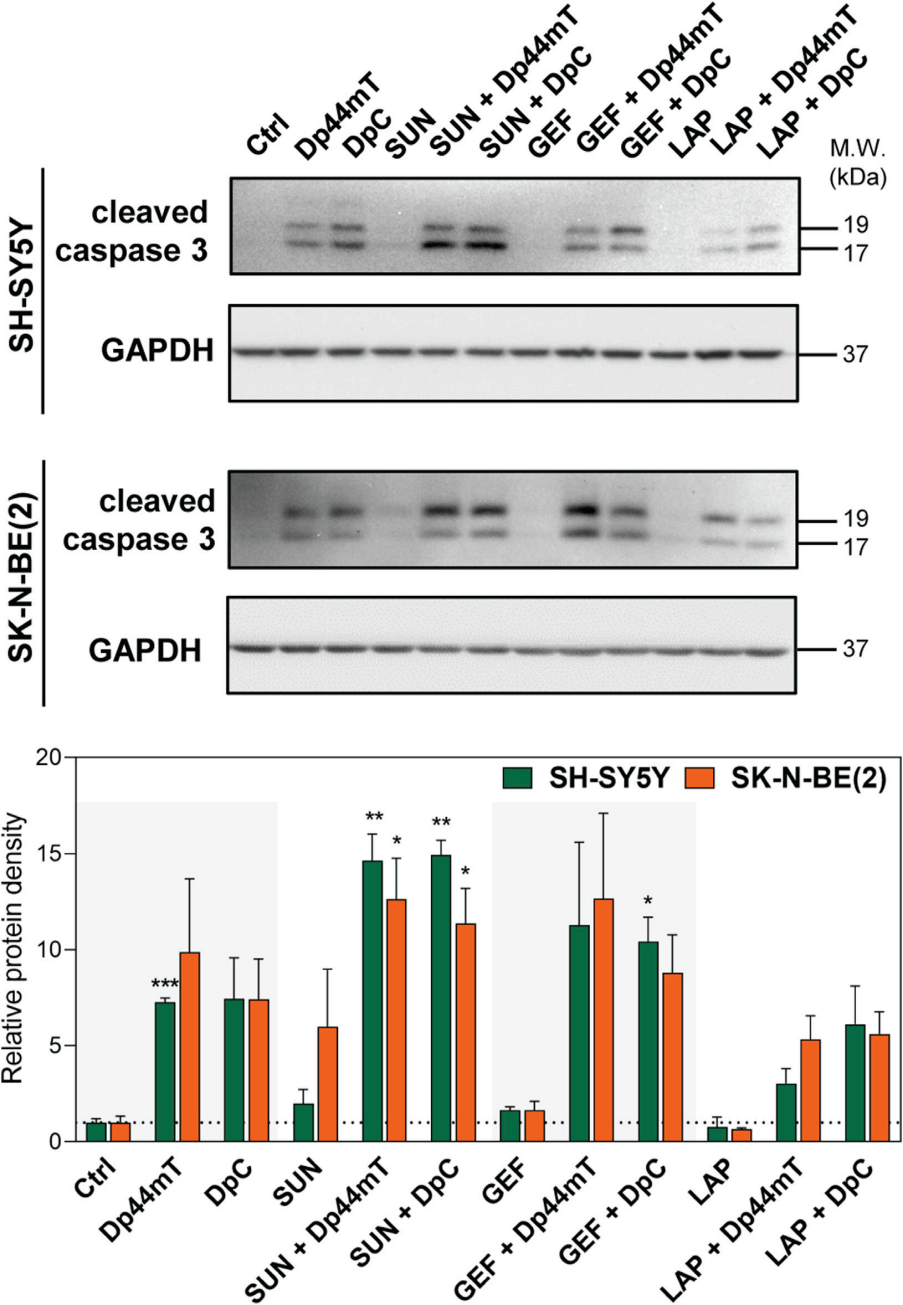
Western blotting of cleaved caspase-3, a marker of apoptosis, in SH-SY5Y and SK-N-BE(2) neuroblastoma cells. The changes in caspase-3 cleavage were detected after 72-hour incubation with either a control medium or a medium containing the drugs, including TKIs (SUN, GEF, or LAP) and thiosemicarbazones (Dp44mT or DpC) alone or in combination. The drugs were used at concentrations of IC_50_ for 72 h as listed in Table 2. Representative blots (left) and their densitometric quantification (right) of three independent experiments are shown. The data are presented as mean ± SD (*n* = 3) normalized to GAPDH that was used as the protein-loading control. *p < 0.05, **p < 0.01, ***p < 0.001 relative to the untreated control samples; the *p* values were evaluated using Welch’s ANOVA followed by Dunnett’s T3 multiple comparisons test. The dotted horizontal line in the graph represents the control levels of cleaved caspase-3.

Since this treatment design was proven to represent the most effective approach, we further analyzed whether the synergistic interactions of the drugs are reflected by an increase in the induction of apoptosis. Due to higher sensitivity to the drugs and a substantial response to combined treatments, SH-SY5Y and SK-N-BE(2) neuroblastoma cells were selected as a model and were treated with thiosemicarbazones and TKIs alone or in combination using the concentrations corresponding to the IC_50_ values. We detected a prominent increase in caspase-3 cleavage in the cells treated with Dp44mT and DpC alone and in combination with TKIs, notably SUN or GEF (Figure 4).

Overall, the results of the experiments performed according to the Simultaneous combined treatment protocol indicated synergistic inhibition of the proliferation of pediatric cancer cells (Figure 3), and induction of apoptosis in tested neuroblastoma cells (Figure 4). Therefore, this treatment design and SH-SY5Y and SK-N-BE(2) neuroblastoma cell lines were selected to further examine the molecular mechanisms of the interactions between thiosemicarbazones and selected TKIs.

### 3.4 DpC downregulates the phosphorylation of the key RTKs in SK-N-BE(2) cells

**FIGURE 5:**
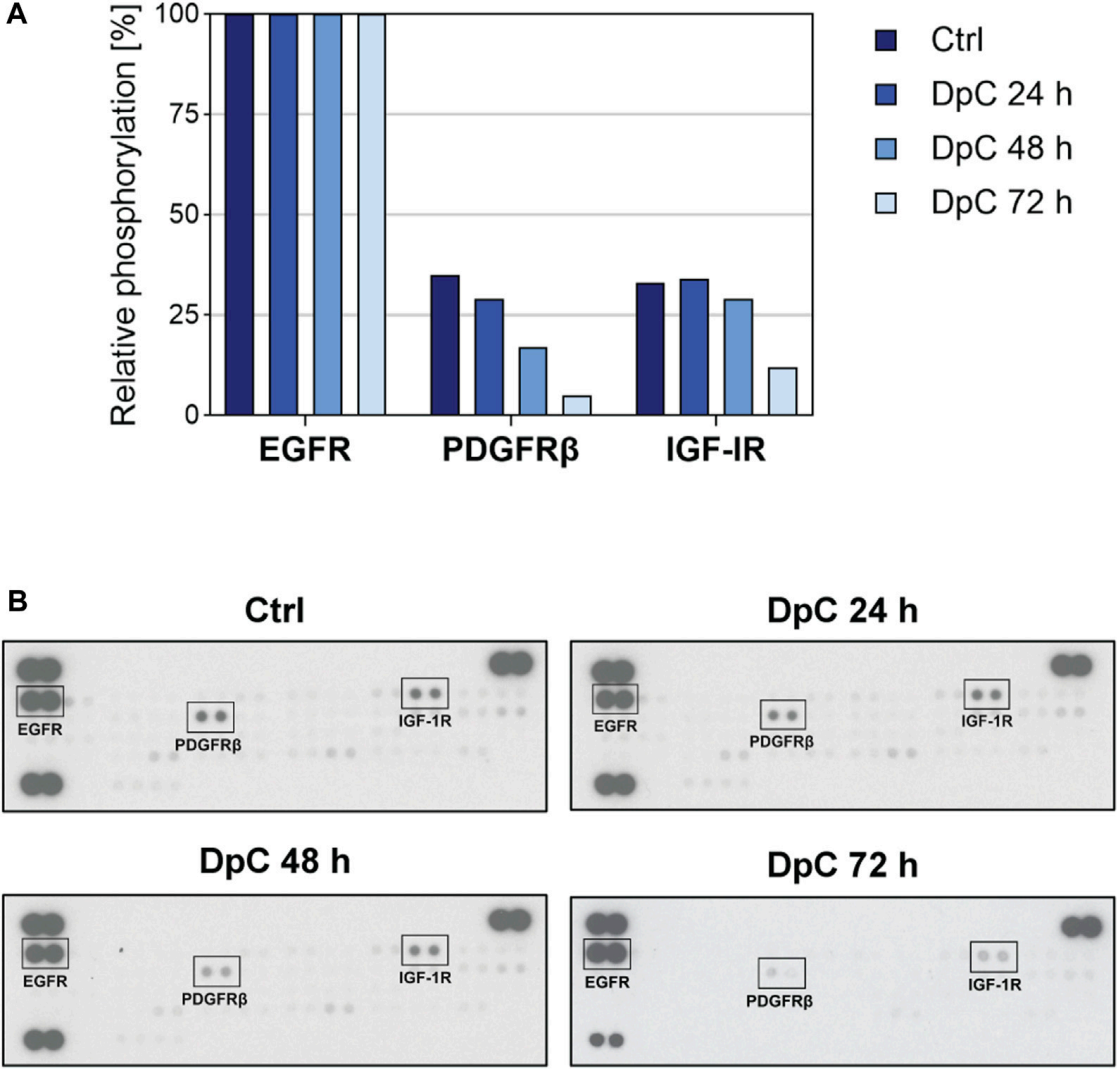
Phospho-RTK array screening in the SK-N-BE(2) neuroblastoma cell line after treatment with DpC. The cells were incubated with a control medium or a medium containing 6.3 nM DpC (the IC_50_ value obtained after 72 h) for 24, 48, and 72 h. **(A)** Evaluation of the relative phosphorylation of selected RTKs (EGFR, PDGFRβ, and IGF-1R). Columns represent the relative phosphorylation of RTKs assessed as described in Materials and Methods. **(B)** The images of the corresponding phospho-RTK arrays of untreated and treated cells were captured on an X-ray film at a constant exposure. Selected RTKs are marked by black rectangles. EGFR, epidermal growth factor receptor; IGF-1R, insulin-like growth factor-1 receptor; PDGFRβ, platelet-derived growth factor receptor β.

Since thiosemicarbazones have been shown to affect various RTKs and the downstream signaling pathways in the cells derived from carcinomas (Dixon et al., 2013; Kovacevic et al., 2013; Lui et al., 2015a; Liu et al., 2015; Kovacevic et al., 2016; Park et al., 2020a; Lim et al., 2020), and in neuroblastoma cells (Guo et al., 2016; Paukovcekova et al., 2020; Macsek et al., 2022), we treated SK-N-BE(2) cells with DpC to identify the potential RTK targets. Human phosphoprotein arrays were used to determine the changes in the phosphorylation of RTKs in SK-N-BE(2) cells after 24-, 48-, and 72-hour incubation with DpC (Figure 5; Supplementary Figure S4).

Thiosemicarbazones have been reported to downregulate EGFR expression and phosphorylation in pancreatic and colon cancer cells (Kovacevic et al., 2016); however, we did not detect any alterations in EGFR activity after treatment with DpC. In contrast, the phosphorylation of IGF-1R and PDGFRβ, which were activated in untreated cells, was markedly decreased during the treatment, and was almost completely abrogated after 72-hour incubation with DpC (Figure 5). The relative phosphorylation levels of other tested RTKs of the panel are shown in Supplementary Figure S4.

Considering the data of the phosphoprotein array screening and previous studies, which have described the modulation of cell signaling by thiosemicarbazones (Dixon et al., 2013; Kovacevic et al., 2016; Paukovcekova et al., 2020; Geleta et al., 2021; Macsek et al., 2022), we further focused on the signaling pathways that could have been affected by both thiosemicarbazones and TKIs selected for the present study.

### 3.5 Combinations of thiosemicarbazones with TKIs reduce phosphorylation and downregulate the key RTKs in neuroblastoma cells

The next part of the present study examined the combined effects of thiosemicarbazones and TKIs on selected RTKs that were shown to be activated in both SH-SY5Y and SK-N-BE(2) cells. These effects included EGFR expression, major sites of EGFR phosphorylation (Tyr1068 and Tyr1148) relevant to EGFR activation, expression of PDGFRβ, Tyr751 phosphorylation of PDGFRβ needed for PI3K activation, and IGF-1R expression.

**FIGURE 6:**
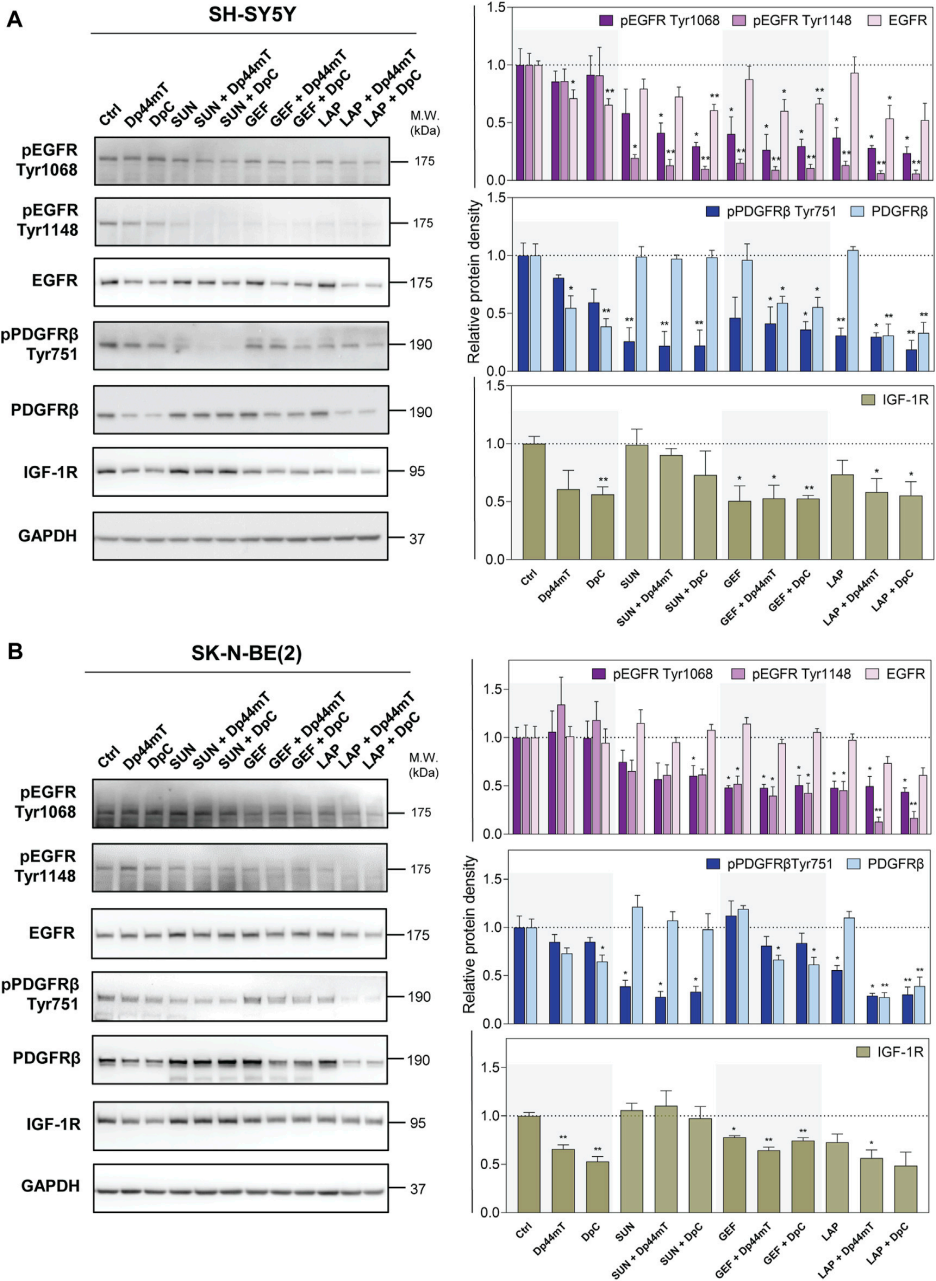
Western blotting of the levels of total and phosphorylated selected RTK proteins, including epidermal growth factor receptor (EGFR), platelet-derived growth factor receptor-β (PDGFRβ), and insulin-like growth factor receptor (IGF-1R). The changes in the levels of phosphorylated EGFR (Tyr1068 and Tyr1148) and PDGFRβ (Tyr751) and of total EGFR, PDGFRβ, and IGF-1R proteins were detected in SH-SY5Y **(A)** and SK-N-BE(2) **(B)** cells after a 72-hour incubation with either a control medium or a medium containing the drugs, including TKIs (SUN, GEF, or LAP) and thiosemicarbazones (Dp44mT or DpC) alone or in combination. The drugs were used at concentrations of IC_50_ for 72 h as listed in Table 2. Representative blots (left) and their densitometric quantification (right) of three independent experiments are shown. The data are presented as mean ± SD (*n* = 3) normalized to GAPDH that was used as the protein-loading control. *p < 0.05, **p < 0.01, ***p < 0.001 relative to the untreated control samples; the *p* values were evaluated using Welch’s ANOVA followed by Dunnett’s T3 multiple comparisons test. The dotted horizontal line in the graph represents the corresponding protein levels detected in the control cells.

As expected, TKIs inhibited the phosphorylation of the corresponding RTKs in both neuroblastoma cell lines; however, we also detected significant off-target inhibitory activity in the case of other tested RTKs (Figure 6). In SH-SY5Y cells, SUN reduced EGFR activation at Tyr1148, and GEF and LAP alone targeted pPDGFRβ (Figure 6A). In SK-N-BE(2) cells, these effects were observed only after LAP treatment (Figure 6B). TKIs did not significantly alter the total EGFR or PDGFRβ levels; however, LAP and especially GEF downregulated IGF-1R in both cell lines (Figure 6).

In agreement with the data of phosphoprotein array analysis, EGFR phosphorylation was not significantly affected in DpC-treated SK-N-BE(2) cells (Figure 6B). The studies of other authors shown EGFR inhibition and/or downregulation by thiosemicarbazones (Liu et al., 2015; Kovacevic et al., 2016; Menezes et al., 2017; Macsek et al., 2022); however, the results of the present study demonstrated a Dp44mT- and DpC-induced decrease in total EGFR only in SH-SY5Y cells (Figure 6A). In contrast, Dp44mT and DpC consistently downregulated PDGFRβ and IGF-1R in both neuroblastoma cell lines (Figure 6).

In cells treated with the drug combinations, pEGFR (Tyr1068 and Tyr1148) was uniformly decreased in both cell lines, and this decrease was detected even in cells treated with the SUN + Dp44mT/DpC combinations (Figure 6). The combination of thiosemicarbazones and TKIs significantly downregulated EGFR expression in SH-SY5Y cells (Figure 6A); however, only a partial EGFR reduction was detected in LAP + Dp44mT/DpC-treated SK-N-BE(2) cells (Figure 6B).

Similarly, inhibition of pPDGFRβ (Tyr751) was detected across all drug combinations in both neuroblastoma cell lines (Figure 6) but was not detected in SK-N-BE(2) cells treated with GEF and thiosemicarbazones (Figure 6B). The levels of the PDGFRβ protein in the cells incubated with the combinations containing SUN remained comparable to the levels in the control cells, whereas the combination of GEF or LAP with thiosemicarbazones resulted in PDGFRβ downregulation in both cell lines (Figure 6). Furthermore, IGF-1R expression was significantly decreased in the cells treated with the latter combinations (Figure 6).

These data showed that the phosphorylation and/or expression of the key pro-oncogenic RTKs was activated in SH-SY5Y and SK-N-BE(2) neuroblastoma cells; specifically EGFR, PDGFRβ, and IGF-1R were inhibited by both thiosemicarbazones and TKIs. Importantly, these effects were more pronounced in the cells treated with the drug combinations, especially after simultaneous incubation with LAP and Dp44mT/DpC, suggesting that the observed synergistic effects were at least to some extent mediated by the blockade of the activity of these crucial RTKs.

### 3.6 Thiosemicarbazones combined with TKIs modulate the signaling of the downstream kinases in neuroblastoma cells

**FIGURE 7:**
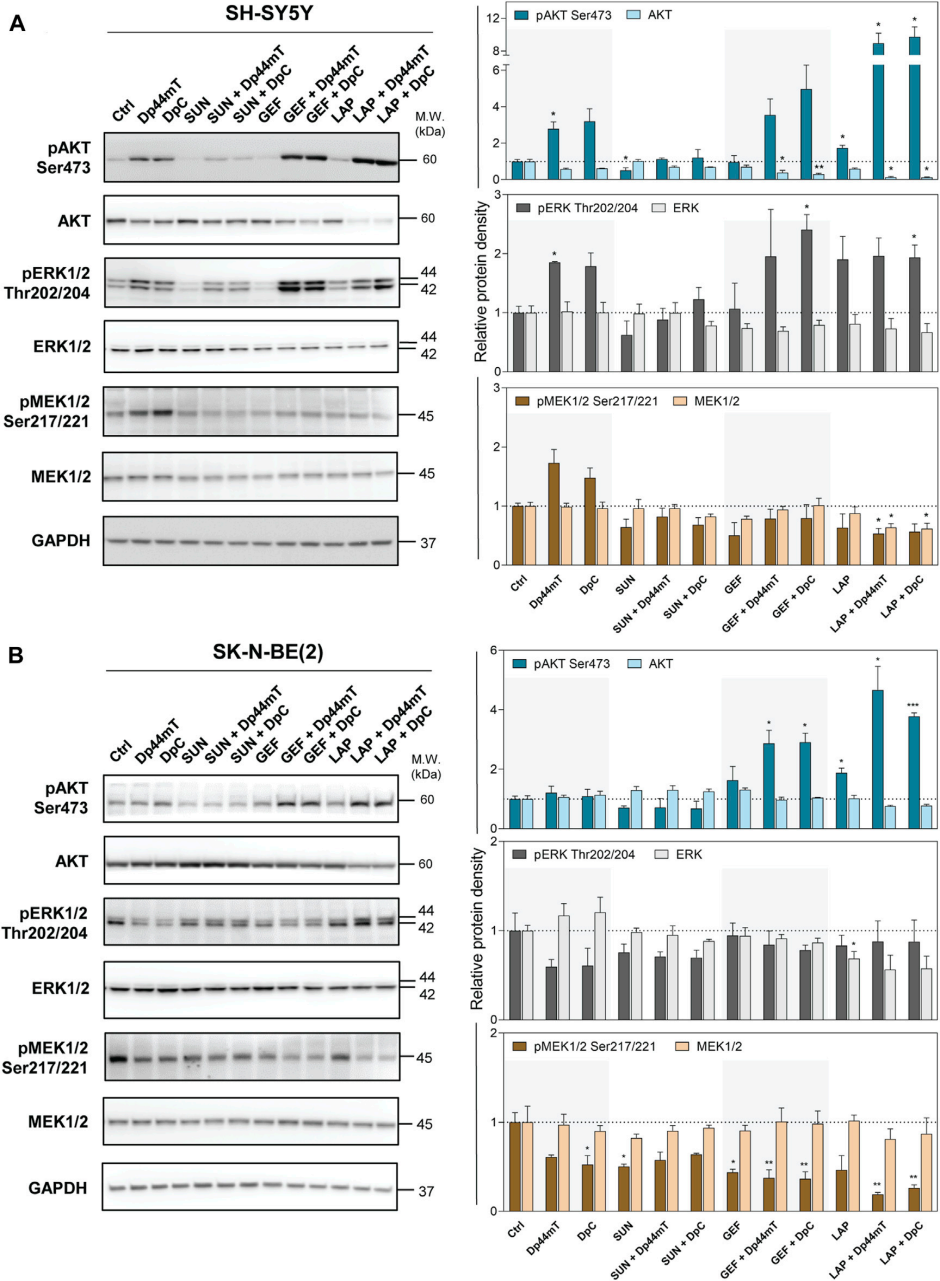
Western blotting of AKT, ERK1/2, and MEK1/2 protein kinases. The changes in the total protein levels and phosphorylation of AKT (Ser473), ERK1/2 (Thr202/204), and MEK1/2 (Ser 217/221) were detected in SH-SY5Y **(A)** and SK-N-BE(2) **(B)** cells after a 72-hour incubation with either a control medium or a medium containing the drugs, including TKIs (SUN, GEF, or LAP) and thiosemicarbazones (Dp44mT or DpC) alone or in combination. The drugs were used at concentrations of IC_50_ for 72 h as listed in Table 2. Representative blots (left) and their densitometric quantification (right) of three independent experiments are shown. The data are presented as mean ± SD (*n* = 3) normalized to GAPDH that was used as the protein-loading control. *p < 0.05, **p < 0.01, ***p < 0.001 relative to the untreated control samples; the *p* values were evaluated using Welch’s ANOVA followed by Dunnett’s T3 multiple comparisons test. The dotted horizontal line in the graph represents the corresponding protein levels detected in the control cells.

Therefore, in the next step, we aimed to determine whether these changes impair the downstream signaling pathways. Since thiosemicarbazones alone have been shown to target PI3K/AKT and MAPK signaling in carcinoma cells (Dixon et al., 2013; Kovacevic et al., 2013, 2016; Lui et al., 2015a; Menezes et al., 2017; Macsek et al., 2022), we assessed the expression and phosphorylation of AKT (Ser473), ERK1/2 (Thr202/204), and MEK1/2 (Ser201/221) after combined treatments of neuroblastoma cells (Figure 7).

Unexpected activation of these downstream kinases was detected after the treatment of SH-SY5Y cells with thiosemicarbazones (Figure 7A). Similar activation of pAKT and pERK1/2 was observed in these cells after treatment with the combinations of thiosemicarbazones with GEF or LAP (Figure 7A). Thiosemicarbazones alone did not influence AKT activation in SK-N-BE(2) cells; however, an increase in pAKT was detected when thiosemicarbazones were combined with GEF or LAP (Figure 7B). Although activation of these kinases was unexpected, other authors have shown similar effects of Dp44mT in prostate cancer cells (Dixon et al., 2013), and we have previously demonstrated the activation of AKT and induction of a stress response in DpC-treated SH-SY5Y cells (Macsek et al., 2022).

In contrast, thiosemicarbazones alone inhibited pERK1/2 and pMEK1/2 in SK-N-BE(2) cells (Figure 7B). Furthermore, a decrease in pMEK1/2 was prominent after treatment of SK-N-BE(2) cells with all drug combinations (Figure 7B). In SH-SY5Y cells, this decrease was detected only after treatment with a combination of LAP and Dp44mT or DpC (Figure 7A).

In summary, combined treatments did not uniformly impair the kinases downstream of analyzed RTKs in the tested neuroblastoma cell lines. In fact, the activation of these kinases may be attributed to other upstream receptors, which were unaffected by the treatments and/or were triggered by a stress response of the cells.

### 3.7 NDRG1 is prominently activated and upregulated after treatment with the combinations of thiosemicarbazones with GEF and LAP in neuroblastoma cells

**FIGURE 8:**
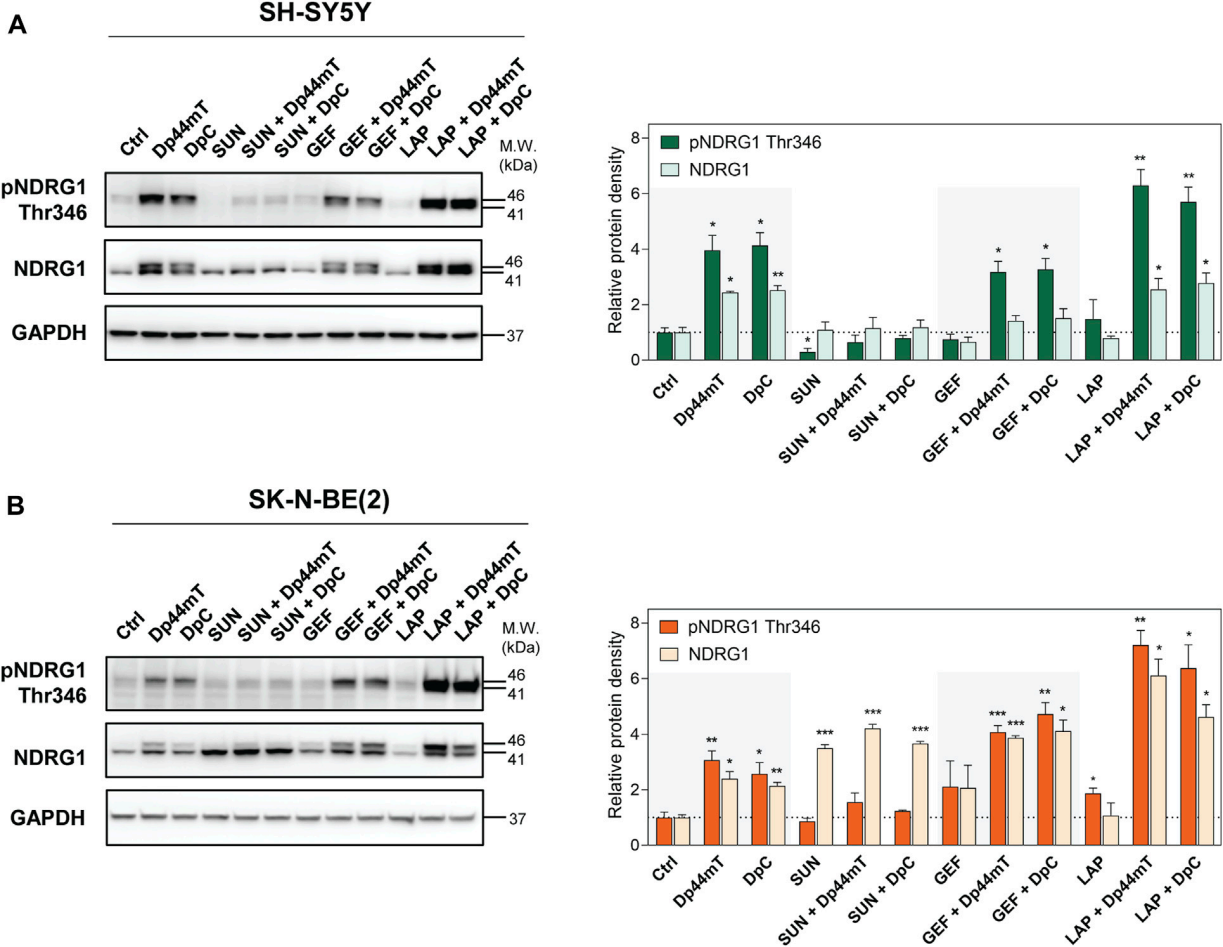
Western blotting of NDRG1. The changes in the levels of phosphorylated NDRG1 (Thr346) and total NDRG1 protein were detected in SH-SY5Y **(A)** and SK-N-BE(2) **(B)** cells after a 72-hour incubation with either a control medium or a medium containing the drugs, including TKIs (SUN, GEF, or LAP) and thiosemicarbazones (Dp44mT or DpC) alone or in combination. The drugs were used at concentrations of IC_50_ for 72 h as listed in Table 2. Representative blots (left) and their densitometric quantification (right) of three independent experiments are shown. The data are presented as mean ± SD (*n* = 3) normalized to GAPDH that was used as the protein-loading control. *p < 0.05, **p < 0.01, ***p < 0.001 relative to the untreated control samples; the *p* values were evaluated using Welch’s ANOVA followed by Dunnett’s T3 multiple comparisons test. The dotted horizontal line in the graph represents the corresponding protein levels detected in the control cells.

NDRG1 has been described as a potent metastasis suppressor in a number of tumors, e.g., colon, prostate, and breast cancers (Bae et al., 2013; Park et al., 2020b). NDRG1 is often upregulated after stress-inducing stimuli (Bae et al., 2013), including cellular iron depletion (Chen et al., 2012; Lane et al., 2013; Lui et al., 2015a; Kovacevic et al., 2016). In fact, NDRG1 has been identified as a target of both Dp44mT and DpC in adult and pediatric tumors (Kovacevic et al., 2016; Menezes et al., 2017; Paukovcekova et al., 2020; Macsek et al., 2022) and implicated in the downregulation of the molecules involved in signal transduction, such as the ErbB family of RTKs and several downstream kinases (Liu et al., 2012; Dixon et al., 2013; Kovacevic et al., 2016; Menezes et al., 2017; Macsek et al., 2022). Our previous studies have shown a prominent upregulation of NDRG1 in the Dp44mT- and DpC-treated cancer cell lines derived from pediatric solid tumors, including neuroblastomas (Paukovcekova et al., 2020; Macsek et al., 2022); thus, we evaluated the NDRG1 levels after combined treatments of SH-SY5Y and SK-N-BE(2) cells (Figure 8). NDRG1 was detected as two closely migrating bands at 41 and 46 kDa, as reported previously (Park et al., 2018, 2020b; Paukovcekova et al., 2020; Macsek et al., 2022).

Similar to other cell lines, Dp44mT and DpC markedly elevated NDRG1 phosphorylation and expression in both SH-SY5Y and SK-N-BE(2) neuroblastoma cells (Figure 8). SUN treatment induced pNDRG1 inhibition in SH-SY5Y cells (Figure 8A); however, it considerably increased total NDRG1 expression in SK-N-BE(2) cells. Moreover, only the lower band at 41 kDa was preferentially upregulated (Figure 8B). In contrast, LAP treatment tended to increase pNDRG1 (Figure 8), although this change was significant only in SK-N-BE(2) cells (Figure 8B).

In both cell lines, the combinations of thiosemicarbazones with SUN reduced NDRG1 phosphorylation compared with Dp44mT- and DpC-treated controls (Figure 8). However, these combined treatments induced the upregulation of total NDRG1 in SK-N-BE(2) cells, and the effect was specifically directed at the 41 kDa band (Figure 8B). In contrast, the combinations of thiosemicarbazones with LAP or GEF markedly elevated pNDRG1 in both cell lines or in SK-N-BE(2) cells, respectively (Figure 8). In SK-N-BE(2) cells, the synergy between thiosemicarbazones and both LAP and GEF was manifested as an apparent upregulation of total NDRG1 expression compared with the effects of these agents alone (Figure 8B).

These data demonstrated that the combinations of thiosemicarbazones, particularly with GEF or LAP, enhanced the efficacy of these agents in upregulating and activating the metastasis suppressor NDRG1 in neuroblastoma cells. These data were in agreement with our initial assessment that revealed strong synergistic interactions of these drugs not only in both neuroblastoma cell lines but also in the tested pediatric solid tumor cell lines in general (Figure 3).

## 4 Discussion

Approximately 30% of RTKs are mutated or overexpressed in malignant diseases, which makes RTKs the key regulators of malignancy in multiple tumor types, including tumors affecting children (Du and Lovly, 2018). The results of our initial screening showed the activation of the key RTKs in the cell lines derived from various pediatric solid tumors, including neuroblastoma, medulloblastoma, osteosarcoma, and rhabdomyosarcoma. Many of these RTKs are known oncoproteins that promote tumorigenesis and tumor progression, making them suitable targets for anticancer therapy (Crose and Linardic, 2010; Yamaoka et al., 2018). Thus, we selected TKIs used in pediatric oncology, specifically SUN, GEF, and LAP, for the present study (Crose and Linardic, 2010; Pollack et al., 2010; Fouladi et al., 2013; Ségaliny et al., 2015).

These TKIs exhibit antiproliferative effects in cancer cells, and these observations were supported by the results of the present study; however, application of these TKIs as monotherapy for anticancer treatment often leads to the development of resistance (Jiao et al., 2018; Krchniakova et al., 2020). Thus, sequential or simultaneous applications of TKIs with other chemotherapeutics have been suggested as a promising approach to establish more effective therapies to overcome drug resistance (Krchniakova et al., 2020). An increase in antitumor efficacy and patient survival has been observed in multiple clinical trials, e.g., using a combination of GEF with carboplatin and pemetrexed in patients with non-small-cell lung carcinoma (Hosomi et al., 2020), of LAP with capecitabine (Cetin et al., 2014) or paclitaxel to treat breast carcinoma (Di Leo et al., 2008), or of SUN with docetaxel in breast carcinoma (Bergh et al., 2012) or gastric carcinoma patients (Yi et al., 2012).

Previous studies have shown that the thiosemicarbazone iron chelators Dp44mT and DpC are potent and selective against multiple adult cancer cell types (Liu et al., 2012; Lovejoy et al., 2012; Dixon et al., 2013; Jansson et al., 2015; Kovacevic et al., 2016; Xu et al., 2018) and cancer cell lines derived from pediatric solid tumors (Guo et al., 2016; Li et al., 2016; Paukovcekova et al., 2020; Macsek et al., 2022). Furthermore, Dp44mT and/or DpC enhance the effect of standard chemotherapeutics, e.g., doxorubicin in various carcinoma cells (Potuckova et al., 2014; Seebacher N. A. et al., 2016), tamoxifen, paclitaxel and 5-fluorouracil in breast carcinoma cells (Potuckova et al., 2014; Maqbool et al., 2020), gemcitabine and cisplatin in lung carcinoma cells (Lovejoy et al., 2012), or celecoxib in pediatric solid tumor cells *in vitro* (Paukovcekova et al., 2020). The present study expanded these findings by analyzing the interactions of Dp44mT and DpC with targeted therapeutics, i.e., TKIs SUN, GEF, and LAP, in the cell lines derived from the most frequent solid tumors in children.

The main goals of the combinational strategies are to achieve a higher efficacy of anticancer therapy while avoiding and/or overcoming drug resistance. The data of the present study suggested that sequential combination treatment designs failed to improve the effects of selected thiosemicarbazones and TKIs; however, simultaneous applications of these drugs resulted in consistent synergy and/or additivity independent of the cancer cell type. Furthermore, this design enabled the use of lower concentrations of these antiproliferative agents, notably Dp44mT and DpC. These results are particularly important because lowering the required drug doses while retaining anticancer efficacy is one of the major aims of combinational therapies (Mokhtari et al., 2017). Furthermore, this approach enables to reduce associated toxicity, which is a crucial aspect of the treatment of the pediatric population (Oeffinger et al., 2007).

TKIs have been developed to attenuate specific RTKs that are frequently dysregulated in cancer cells (Kannaiyan and Mahadevan, 2018). In addition to the inhibition of the corresponding targets, we also identified interesting off-target effects of GEF and LAP on PDGFRβ activation and the effect of SUN on EGFR phosphorylation (at the Tyr1148 residue) in both SH-SY5Y and SK-N-BE(2) neuroblastoma cell lines, which served as the models for detailed analyses of the molecular effects of combined treatments. There are no studies that have focused on this type of activity of GEF or LAP; however, the multikinase inhibitor SUN has been previously shown to target a number of unconventional RTKs, including EGFR, FGFR, TrkA, and TrkB, in neuroblastoma cells (Calero et al., 2014).

In contrast, thiosemicarbazones used in the present study target cancer cells via multiple mechanisms (Lui et al., 2015a). The antiproliferative activity of these derivates is attributed to iron depletion, which subsequently modulates the regulation of cell cycle (Dixon et al., 2013; Lui et al., 2015b), key signaling pathways (Dixon et al., 2013; Kovacevic et al., 2013, 2016; Menezes et al., 2017; Park et al., 2020a; Macsek et al., 2022), apoptosis (Zhou et al., 2020), and autophagy (Gutierrez et al., 2014). The results of the present study indicated that a combination of thiosemicarbazones with TKIs affected critical oncogenic RTKs and/or downstream targets, leading to the synergistic/additive interactions observed in the tested cell types.

Thiosemicarbazones have been demonstrated to inhibit the expression and activation of the ErbB family of receptors in response to EGF in pancreatic carcinoma cells *in vitro* and *in vivo* (Kovacevic et al., 2016; Menezes et al., 2017). A potent metastasis suppressor, NDRG1, has been proposed as the key regulator of these effects (Kovacevic et al., 2016; Menezes et al., 2017). Phosphorylation of NDRG1 is crucial for a number of physiological events, e.g., T-cell clonal anergy or cell division (Park et al., 2020b), and the anticancer effects of NDRG1 have been implicated in multiple cancers (Bae et al., 2013; Park et al., 2018, 2020b; Paukovcekova et al., 2020; Macsek et al., 2022). Furthermore, NDRG1 is modulated via cellular iron levels (Lui et al., 2015a) and is thus upregulated by the chelators, such as Dp44mT and DpC (Chen et al., 2012; Lane et al., 2013; Lui et al., 2015a; Kovacevic et al., 2016; Menezes et al., 2017). We have previously demonstrated the upregulation of NDRG1 by thiosemicarbazones and the anticancer effects of NDRG1 in cancer cells derived from pediatric solid tumors, including neuroblastomas (Paukovcekova et al., 2020; Macsek et al., 2022). As suggested for other cancer types (Kovacevic et al., 2016; Menezes et al., 2017; Macsek et al., 2022), a thiosemicarbazone-mediated decrease in EGFR observed in SH-SY5Y cells in the present study may be attributed to NDRG1 upregulation.

In addition to marked NDRG1 activation in the cells treated with thiosemicarbazones alone, these effects were also detected when thiosemicarbazones were combined with GEF or LAP in both neuroblastoma cell lines. Interestingly, a similar effect was not detected in SUN-treated cells (Figure 8), which may be due to the multikinase inhibitory activity of SUN (Calero et al., 2014). Of note, SUN has been shown to target AKT (Calero et al., 2014), which is one of the activators of NDRG1 (Park et al., 2020b).

In addition to EGFR downregulation, we observed intriguing effects of both Dp44mT and DpC on other tested RTKs, including PDGFRβ and IGF-1R (Figure 6). Although RTKs are presumed to form dimers with the partners of the same RTK family, the formation of the cross-family dimers has also been reported, especially in the case of EGFR (Kennedy et al., 2016). These dimers include EGFR-IGF-1R (Ahmad et al., 2004) or EGFR-PDGFRβ heterodimers (Saito et al., 2001), and their formation may lead to the concomitant degradation of both dimer components, potentially contributing to a decrease in these RTKs after thiosemicarbazone treatment demonstrated in the present study. Moreover, NDRG1 has been implicated in RTK degradation (Kovacevic et al., 2016; Menezes et al., 2019b; Park et al., 2020a). The activity and signaling of multiple growth factor receptors, including EGFR, PDGFRβ, and IGF-1R, is modulated via c-Src (Bromann et al., 2004; Amanchy et al., 2009; Liu et al., 2015), which is one of multiple NDRG1 targets (Liu et al., 2015). Similarly, NDRG1 has been recently shown to induce the expression of proteins involved in the degradation of IGF-1R. Multiubiquitination and subsequent degradation of IGF-1R upon ligand binding is mediated by E3 ubiquitin ligase NEDD4 in complex with the Grb10 adaptor protein, which acts as a bridge between NEDD4 and IGF-1R (Vecchione et al., 2003; Monami et al., 2008). Interestingly, NEDD4-like E3 ubiquitin ligase (NEDD4L) and Grb10 have been identified as the molecular targets of NDRG1 (Zhao et al., 2011; Kovacevic et al., 2013).

NDRG1 overexpression has also been shown to inhibit the downstream targets of EGFR signaling, e.g., MEK1/2 or ERK1/2, in pancreatic and prostate carcinoma cells (Dixon et al., 2013; Kovacevic et al., 2013, 2016). However, the data of the present study indicated that the treatment with thiosemicarbazones resulted in significantly upregulated phosphorylation of AKT, ERK1/2, and MEK1/2 kinases in SH-SY5Y cells. Enhanced kinase activation in the cells incubated with Dp44mT or DpC has been detected previously (Dixon et al., 2013; Macsek et al., 2022) and may be a result of a pro-survival response of the cells to stress stimuli induced by drug treatment (Dixon et al., 2013; Macsek et al., 2022). However, a different response of kinase signaling was detected in SK-N-BE(2) cells. Although both cell lines included in the present study are derived from neuroblastomas, each of these cell lines represent different neuroblastoma cell phenotypes (Ross et al., 2003). The heterogeneity of the cell populations is a distinctive feature of neuroblastomas, and this heterogeneity may be responsible for the discrepancies between the neuroblastoma cell lines detected in the present study. SH-SY5Y cells manifest the characteristics of N-type (neuroblastic/neuroendocrine precursors) neuroblastoma cells, and SK-N-BE(2) cells manifest a stem cell phenotype of I-type cells (Ross et al., 2003). Furthermore, the amplification of *MYCN*, which is a major prognostic marker for neuroblastoma, was detected only in SK-N-BE(2) cells, and the N-myc protein is known to repress NDRG1 (Li and Kretzner, 2003). Despite these differences, a combination of thiosemicarbazones with TKIs induced uniformly synergistic and/or additive interactions in both neuroblastoma cell lines and increased apoptosis, especially after the combined treatments.

In conclusion, the combined treatments with thiosemicarbazones and TKIs have substantial synergistic potential for anticancer therapies of pediatric solid tumors. Simultaneous administration of the drugs was identified as the most potent approach for application of the combinations of thiosemicarbazones Dp44mT and DpC with TKIs SUN, GEF, and LAP in all tested cell lines. TKIs inhibited the activation of the corresponding RTKs; however, both thiosemicarbazones decreased the expression of RTKs, including EGFR and novel targets PDGFRβ and IGF-1R identified in the present study. The downregulation of NDRG1 mRNA expression has been shown to be associated with poor prognosis of neuroblastoma patients (Matsushita et al., 2013). Considering this finding and the data obtained in the present and previous studies (Paukovcekova et al., 2020; Macsek et al., 2022), we suggest that the upregulation of NDRG1, which is detected after combined treatment with thiosemicarbazones and GEF or LAP, presents a promising strategy for neuroblastoma treatment. The exact molecular functions of NDRG1 in the cells after combined treatment require further investigation; however, the present study provided a valid rationale for combined therapy of pediatric solid tumors using iron-chelating agents together with TKIs, especially with GEF or LAP.

## Data Availability

The data that support the findings of the present study are available from the corresponding authors upon reasonable request.

## Abbreviations

AKT/PI3K, protein kinase B/phosphatidylinositol-3-kinase; BSA, bovine serum albumin; CI, combination index; DMEM, Dulbecco's modified Eagle's medium; DMSO, dimethyl sulfoxide; Dp44mT, di-2-pyridylketone-4,4-dimethyl-3-thiosemicarbazone; DpC, di-2-pyridylketone-4-cyclohexyl-4-methyl-3-thiosemicarbazone; EGFR, epidermal growth factor receptor; FCS, fetal calf serum; GAPDH, glyceraldehyde-3-phosphate dehydrogenase; GEF, gefitinib; IGF-1R, insulin-like growth factor-1 receptor; InsR, insulin receptor; LAP, lapatinib; MDR, multidrug resistance; MTT, 3-[4,5-dimethylthiazol-2-yl]-2,5-diphenyltetrazolium bromide; NDRG1, N-myc downstream regulated gene-1; NEDD4L, NEDD4-like E3 ubiquitin ligase; NFDM, nonfat dry milk; PBS, phosphate buffered saline; PDGFR, platelet-derived growth factor receptor; Pgp, P-glycoprotein; ROS, reactive oxygen species; RTK, receptor tyrosine kinase; SD, standard deviation; SUN, sunitinib; TKI, tyrosine kinase inhibitor; VAL, valspodar; VEGFR, vascular endothelial growth factor receptor.
