# Supplementary material for: Thiosemicarbazones and selected tyrosine kinase inhibitors synergize in pediatric solid tumors: NDRG1 upregulation and impaired prosurvival signaling in neuroblastoma cells
Source: Front Pharmacol. 2022 Sep 7;13:976955. doi: 10.3389/fphar.2022.976955 (PMC9490180; doi:10.3389/fphar.2022.976955)

Supplementary Material

# Supplementary Figures and Tables

## Supplementary Tables

Supplementary Table 1: Quantitative assessment of the antiproliferative activity of the combination of thiosemicarbazones (Dp44mT and DpC) with TKIs (SUN, GEF, and LAP) in the cell lines derived from pediatric solid tumors. The data were obtained using the MTT proliferation assays after incubation at 37°C with each drug combination based on the corresponding experimental design (Pretreatment I; Pretreatment I + Valspodar; Pretreatment II; and Simultaneous treatment). A 1:1 ratio of the drugs was used for the combined treatments according to all design schemes. The combination index (CI) values were calculated using the Chou Talalay method using CalcuSyn Software. The CI values indicate synergistic (CI < 0.9; green), additive (CI: 0.9–1.1; yellow), or antagonistic (CI > 1.1; orange) activity of the tested drugs. The results are presented as the mean ± SD (n=3).

| **Cell line** | **TKI** | **Thiosemi-carbazone** | | **Pretreatment I** | | **Pretreatment I**  **+ Valspodar** | | | | **Pretreatment II** | | | | | **Simultaneous treatment** | | |
| --- | --- | --- | --- | --- | --- | --- | --- | --- | --- | --- | --- | --- | --- | --- | --- | --- | --- |
|  |  |  |  | **CI** | **SD** | **CI** | | **SD** | | **CI** | | **SD** | | **CI** | | **SD** | |
| **DAOY** | **SUN** | **Dp44mT** | | **0.918** | 0.090 | **1.062** | | 0.096 | | **-** | | | | | **0.789** | | 0.094 |
|  |  | **DpC** | | **0.827** | 0.217 | **0.934** | | 0.028 | |  |  |  |  |  | **0.646** | | 0.035 |
|  | **GEF** | **Dp44mT** | | **0.643** | 0.034 | **0.505** | | 0.145 | |  |  |  |  |  | **0.635** | | 0.059 |
|  |  | **DpC** | | **0.669** | 0.193 | **0.703** | | 0.041 | |  |  |  |  |  | **0.610** | | 0.104 |
|  | **LAP** | **Dp44mT** | | **0.416** | 0.186 | **0.502** | | 0.133 | |  |  |  |  |  | **0.405** | | 0.047 |
|  |  | **DpC** | | **0.572** | 0.079 | **0.339** | | 0.124 | |  |  |  |  |  | **0.542** | | 0.067 |
| **SH-SY5Y** | **SUN** | **Dp44mT** | | **1.680** | 0.081 | **1.277** | | 0.019 | | **0.863** | | | 0.180 | | **0.751** | | 0.021 |
|  |  | **DpC** | | **1.632** | 0.126 | **1.303** | | 0.072 | | **0.904** | | | 0.106 | | **0.483** | | 0.048 |
|  | **GEF** | **Dp44mT** | | **0.948** | 0.020 | **0.957** | | 0.047 | | **0.983** | | | 0.156 | | **0.563** | | 0.094 |
|  |  | **DpC** | | **1.084** | 0.132 | **1.062** | | 0.096 | | **0.632** | | | 0.051 | | **0.659** | | 0.018 |
|  | **LAP** | **Dp44mT** | | **1.294** | 0.132 | **0.665** | | 0.111 | | **1.389** | | | 0.069 | | **0.413** | | 0.095 |
|  |  | **DpC** | | **1.393** | 0.046 | **0.501** | | 0.103 | | **1.821** | | | 0.245 | | **0.695** | | 0.104 |
| **SK-N-BE(2)** | **SUN** | **Dp44mT** | | **1.144** | 0.015 | **1.000** | | 0.061 | | **0.779** | | | 0.062 | | **0.910** | | 0.110 |
|  |  | **DpC** | | **1.593** | 0.258 | **1.385** | | 0.130 | | **0.806** | | | 0.073 | | **0.762** | | 0.106 |
|  | **GEF** | **Dp44mT** | | **1.142** | 0.104 | **1.281** | | 0.064 | | **0.792** | | | 0.140 | | **0.640** | | 0.067 |
|  |  | **DpC** | | **1.086** | 0.082 | **0.859** | | 0.087 | | **0.699** | | | 0.101 | | **0.410** | | 0.086 |
|  | **LAP** | **Dp44mT** | | **0.965** | 0.111 | **0.819** | | 0.039 | | **0.615** | | | 0.123 | | **0.432** | | 0.101 |
|  |  | **DpC** | | **1.414** | 0.281 | **1.282** | | 0.134 | | **0.793** | | | 0.098 | | **0.645** | | 0.021 |
| **RD** | **SUN** | **Dp44mT** | | **1.474** | 0.105 | **1.492** | | 0.140 | | **-** | | | | | **1.190** | | 0.066 |
|  |  | **DpC** | | **1.256** | 0.094 | **1.593** | | 0.090 | |  |  |  |  |  | **1.093** | | 0.058 |
|  | **GEF** | **Dp44mT** | | **1.820** | 0.193 | **1.279** | | 0.125 | |  |  |  |  |  | **0.902** | | 0.063 |
|  |  | **DpC** | | **1.199** | 0.117 | **0.869** | | 0.162 | |  |  |  |  |  | **0.632** | | 0.048 |
|  | **LAP** | **Dp44mT** | | **2.430** | 0.270 | **3.028** | | 0.161 | |  |  |  |  |  | **0.626** | | 0.096 |
|  |  | **DpC** | | **2.055** | 0.173 | **1.872** | | 0.107 | |  |  |  |  |  | **1.584** | | 0.091 |
| **Saos-2** | **SUN** | **Dp44mT** | | **1.433** | 0.241 | **1.549** | | 0.097 | | **-** | | | | | **0.467** | | 0.040 |
|  |  | **DpC** | | **1.342** | 0.210 | **1.688** | | 0.071 | |  |  |  |  |  | **0.717** | | 0.056 |
|  | **GEF** | **Dp44mT** | | **1.235** | 0.157 | **2.330** | | 0.100 | |  |  |  |  |  | **0.800** | | 0.063 |
|  |  | **DpC** | | **0.701** | 0.139 | **1.134** | | 0.055 | |  |  |  |  |  | **0.840** | | 0.089 |
|  | **LAP** | **Dp44mT** | | **1.596** | 0.083 | **1.839** | | 0.132 | |  |  |  |  |  | **0.474** | | 0.107 |
|  |  | **DpC** | | **2.077** | 0.295 | **0.963** | | 0.055 | |  |  |  |  |  | **0.385** | | 0.091 |
| **Categories of Interactions** | | | | | | | | | | | | | | | | | |
|  | 0.10–0.30 | | Strong synergism | | | |  | | 0.91–1.10 | | Nearly additive | | | | | | |
|  | 0.31–0.70 | | Synergism | | | |  | | 1.11–1.20 | | Slight antagonism | | | | | | |
|  | 0.71–0.85 | | Moderate synergism | | | |  | | 1.21–1.45 | | Moderate antagonism | | | | | | |
|  | 0.86–0.90 | | Slight synergism | | | |  | | 1.46–3.30 | | Antagonism | | | | | | |

## Supplementary Figures

***
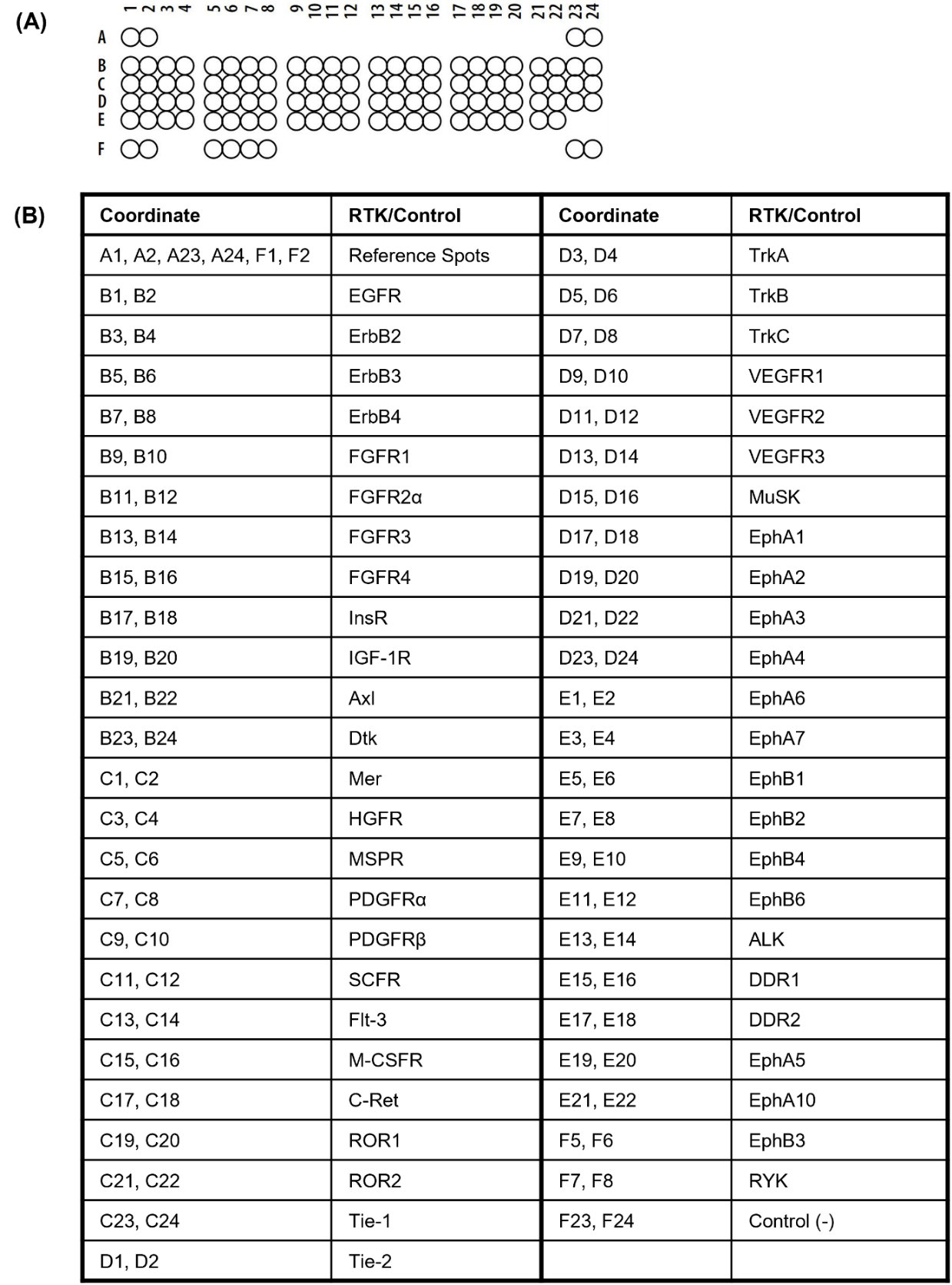
***

Figure S1: (A) The coordinates of the phosphoprotein arrays and (B) 49 RTKs tested using the Proteome Profiler™ Human Phospho-RTK array kit (R&D Systems).


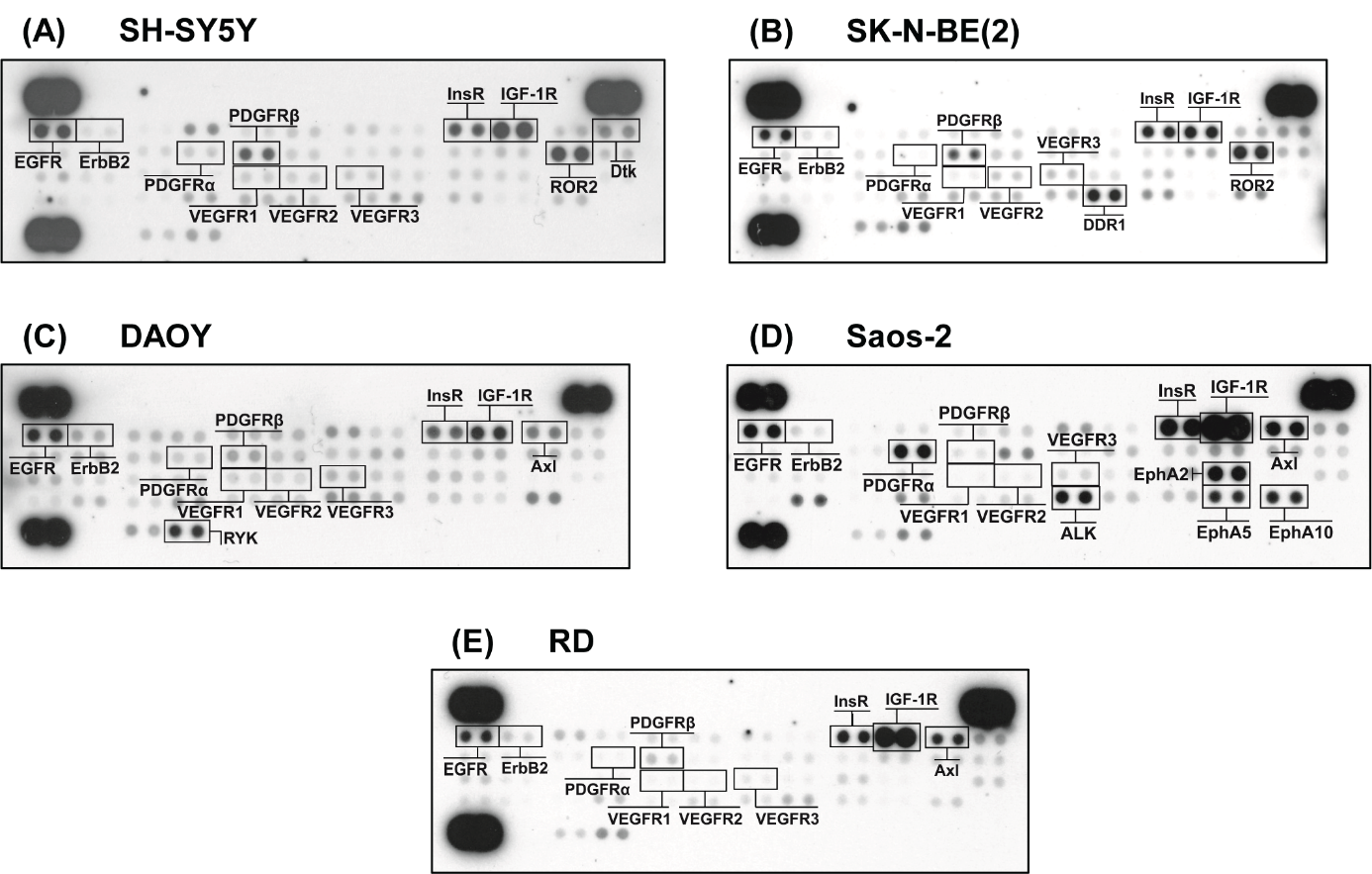


**Supplementary Figure 2:** Array images corresponding to RTK phosphorylation shown in Figure 2, including (A) SH-SY5Y neuroblastoma; (B) SK-N-BE(2) neuroblastoma; (C) DAOY medulloblastoma; (D) Saos-2 osteosarcoma; and (E) RD rhabdomyosarcoma cells. The array images were captured using X-ray film and are shown for each cell line. Black rectangles indicate the targets of TKIs used in the present study (GEF: EGFR; LAP: EGFR and ErbB2; SUN: VEGFRs and PDGFRs) and other highly phosphorylated RTKs detected in each cancer cell type. Abbreviations: DDR1, discoidin domain receptor 1; EGFR, epidermal growth factor receptor; IGF-1R, insulin-like growth factor-1 receptor; InsR, insulin receptor; PDGFR, platelet-derived growth factor receptor; ROR2, RTK-like orphan receptor 2; VEGFR, vascular endothelial growth factor receptor.


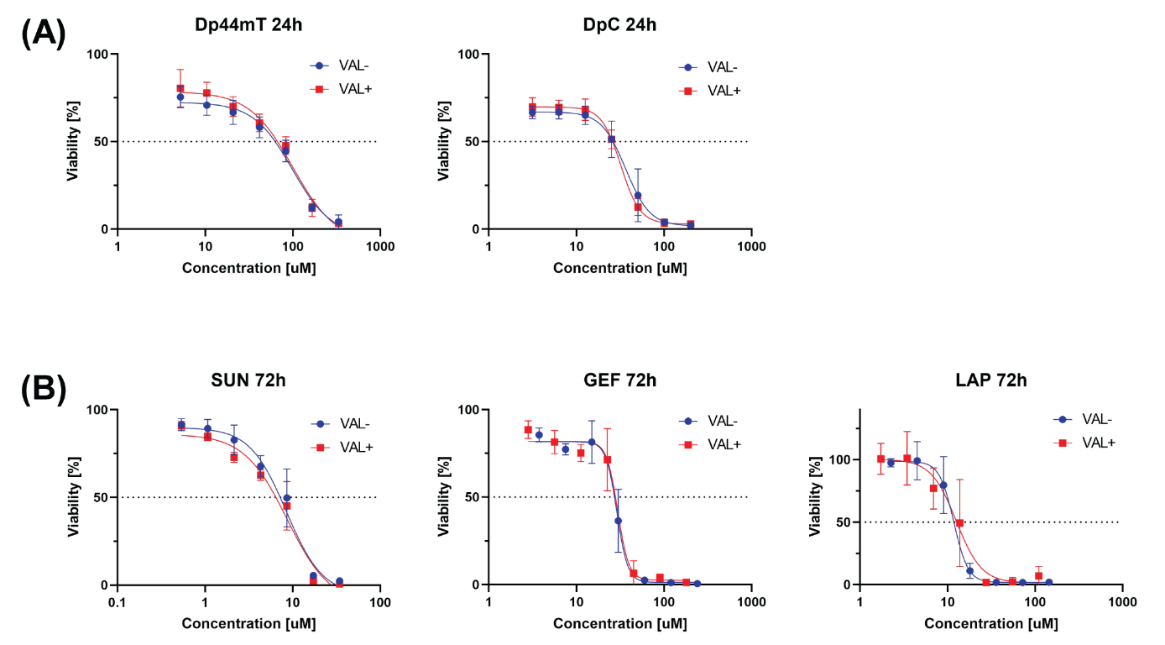


**Supplementary Figure 3:** Comparison of the drug concentrations used for the treatments of Pgp-expressing SH-SY5Y neuroblastoma cells performed according to the Pretreatment I and Pretreatment I + Valspodar strategies: (A) 24-hour incubation with thiosemicarbazones Dp44mT and DpC and (B) 72-hour incubation with TKIs sunitinib (SUN), gefitinib (GEF), and lapatinib (LAP) (n=3). The assays were performed either without Valspodar (VAL(-); blue) or with 0.2 µM Valspodar (VAL(+); red). No significant differences between the VAL(-) and VAL(+) conditions were detected in the case of tested drugs.


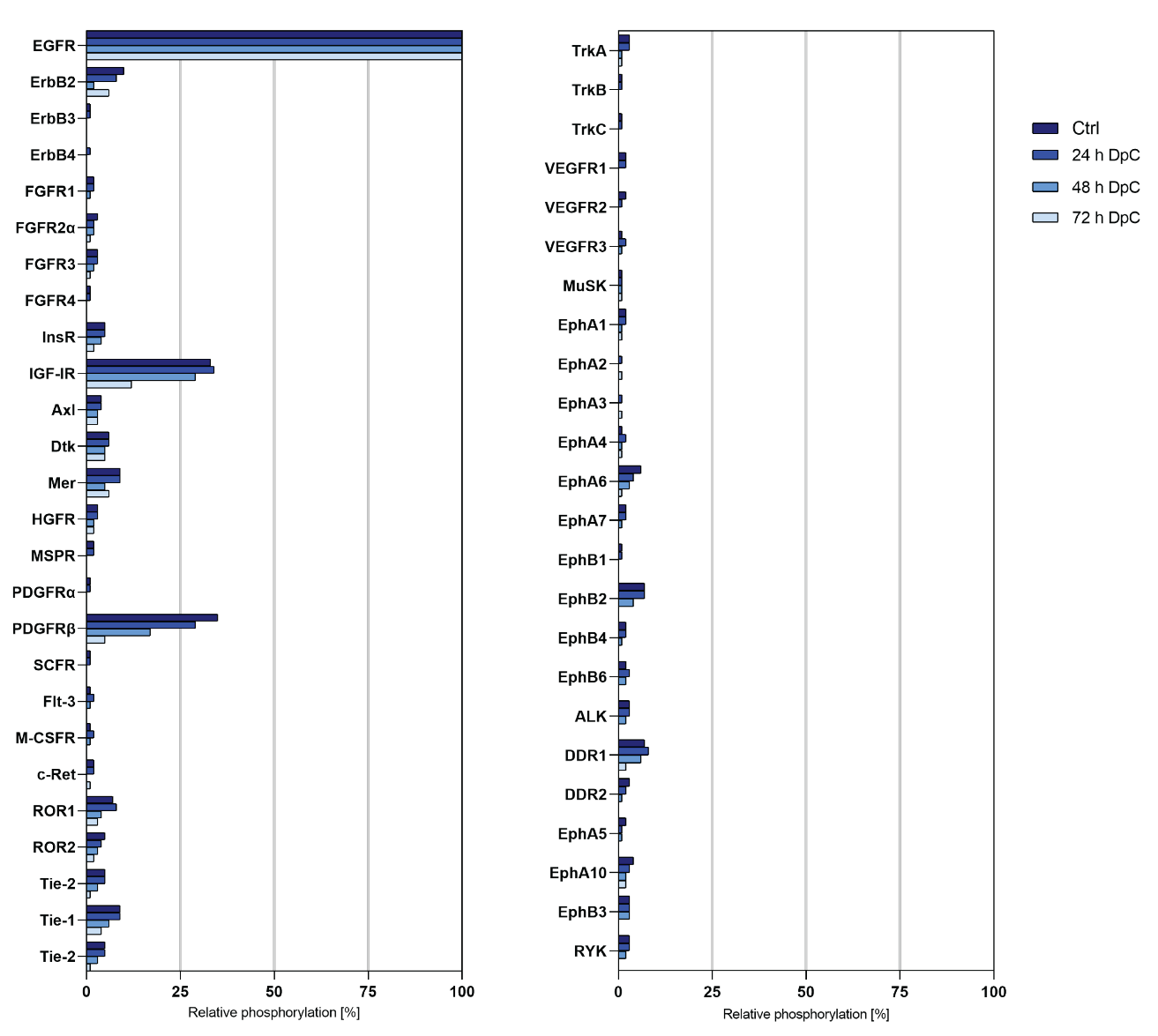


**Supplementary Figure 4:** Phospho-RTK array screening after the incubation of SK-N-BE(2) neuroblastoma cells with a control medium or a medium containing DpC (6.3 nM; IC_50_/72 h). Evaluation of the relative phosphorylation of 49 tested RTKs after 24-, 48-, and 72-hour treatments. Columns represent the levels of relative phosphorylation of RTKs assessed as described in Materials and Methods.

# Supplementary data

All replicates of uncropped Western blotting images used in the analyses.

**Supplementary Data 1:** Uncropped Western blot images of p-EGFR (Tyr1068, Tyr1148) and EGFR immunodetection in SH-SY5Y cell line. GAPDH or α-tubulin served as loading control (see Supplementary Data 12 and 14) and Coomassie stained membranes are included to show loading for respective membranes. The experiments were repeated 3 times. * blots used in Figure 6A. 1: Ctrl; 2: Dp44mT; 3: DpC; 4: SUN; 5: SUN+Dp44mT; 6: SUN+DpC; 7: GEF; 8: GEF+Dp44mT; 9: GEF+DpC; 10: LAP; 11: LAP+Dp44mT; 12: LAP+DpC.


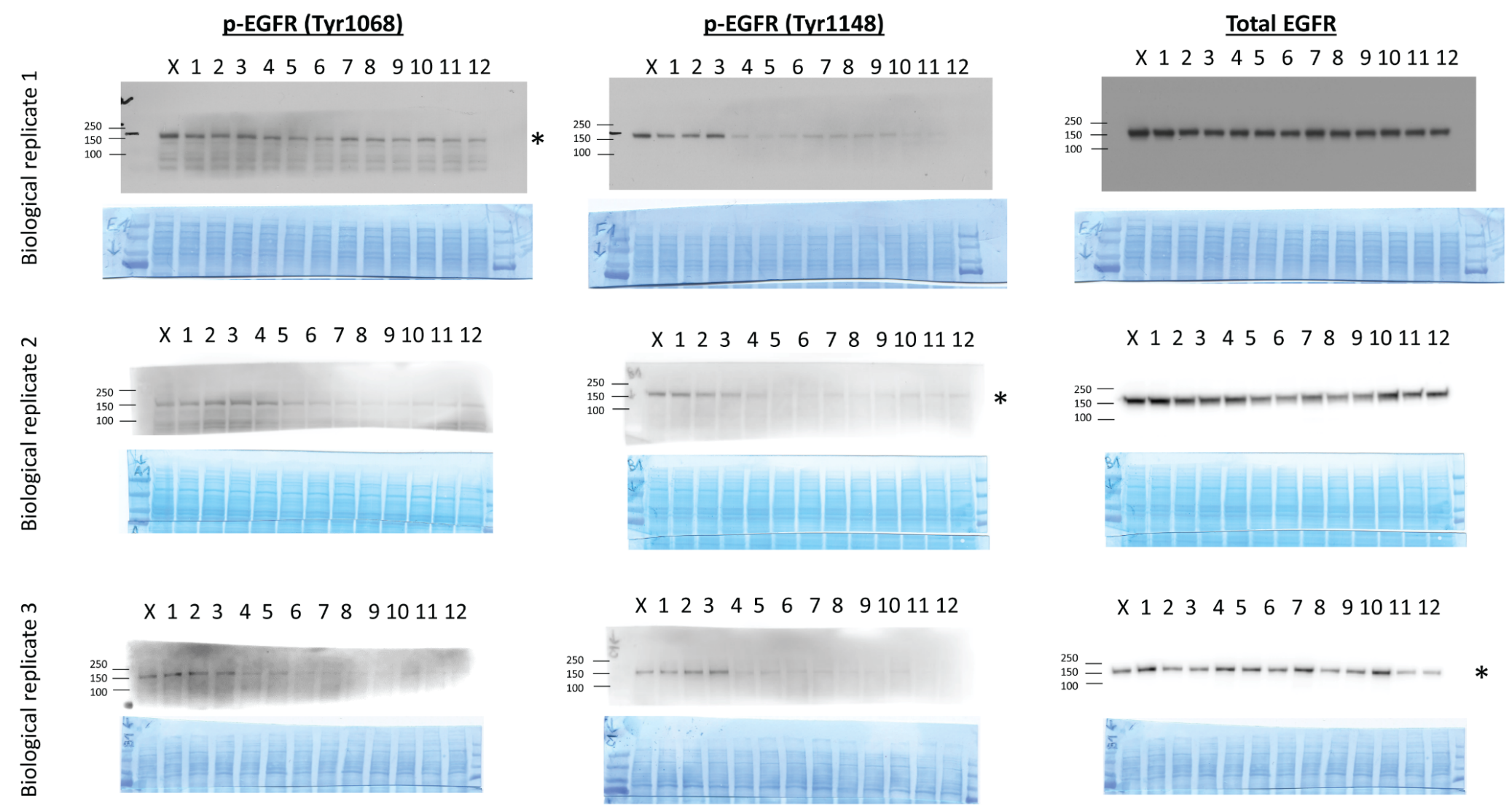


**Supplementary Data 2:** Uncropped Western blot images of p-PDGFRβ (Tyr751), PDGFRβ and IGF-1R immunodetection in SH-SY5Y cell line. GAPDH or α-tubulin served as loading control (see Supplementary Data 12 and 14) and Coomassie stained membranes are included to show loading for respective membranes. The experiments were repeated 3 times. * blots used in Figure 6A. 1: Ctrl; 2: Dp44mT; 3: DpC; 4: SUN; 5: SUN+Dp44mT; 6: SUN+DpC; 7: GEF; 8: GEF+Dp44mT; 9: GEF+DpC; 10: LAP; 11: LAP+Dp44mT; 12: LAP+DpC.


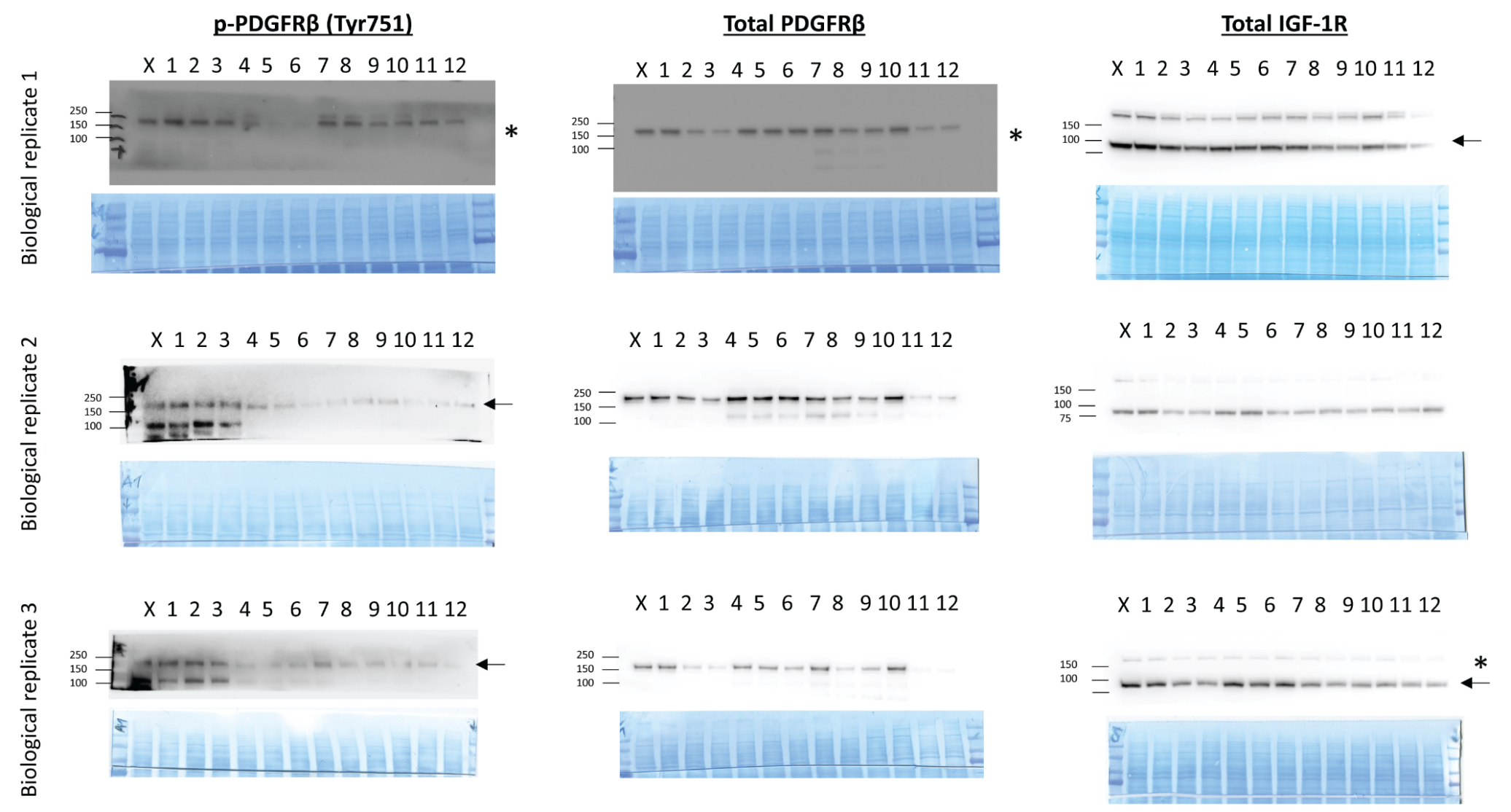


**Supplementary Data 3:** Uncropped Western blot images of p-EGFR (Tyr1068, Tyr1148) and EGFR immunodetection in SK-N-BE(2) cell line. GAPDH or α-tubulin served as loading control (see Supplementary Data 13 and 14) and Coomassie stained membranes are included to show loading for respective membranes. The experiments were repeated 3 times. * blots used in Figure 6B. 1: Ctrl; 2: Dp44mT; 3: DpC; 4: SUN; 5: SUN+Dp44mT; 6: SUN+DpC; 7: GEF; 8: GEF+Dp44mT; 9: GEF+DpC; 10: LAP; 11: LAP+Dp44mT; 12: LAP+DpC.


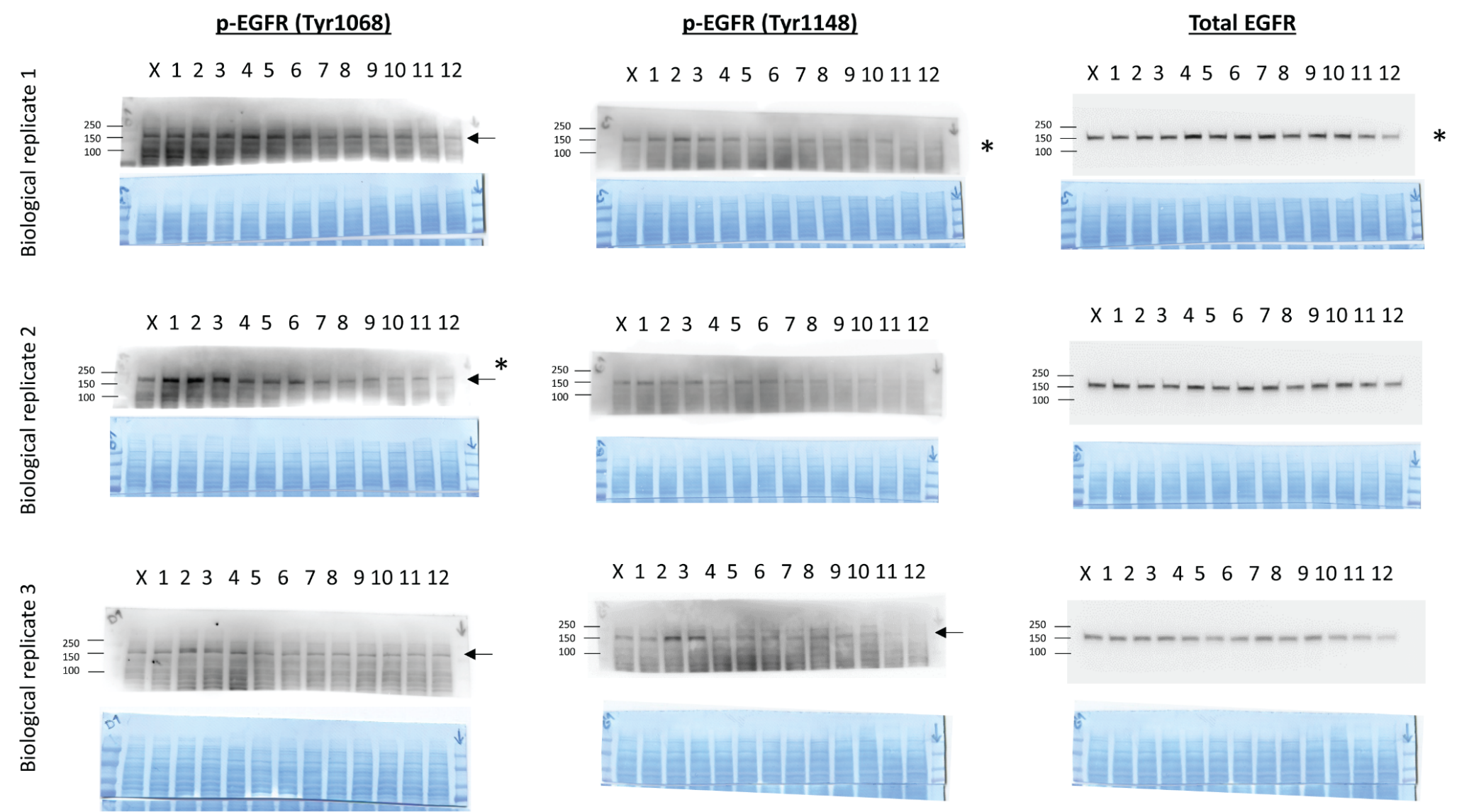


**Supplementary Data 4:** Uncropped Western blot images of p-PDGFRβ (Tyr751), PDGFRβ and IGF-1R immunodetection in SK-N-BE(2) cell line. GAPDH or α-tubulin served as loading control (see Supplementary Data 13 and 14) and Coomassie stained membranes are included to show loading for respective membranes. The experiments were repeated 3 times. * blots used in Figure 6B. 1: Ctrl; 2: Dp44mT; 3: DpC; 4: SUN; 5: SUN+Dp44mT; 6: SUN+DpC; 7: GEF; 8: GEF+Dp44mT; 9: GEF+DpC; 10: LAP; 11: LAP+Dp44mT; 12: LAP+DpC.


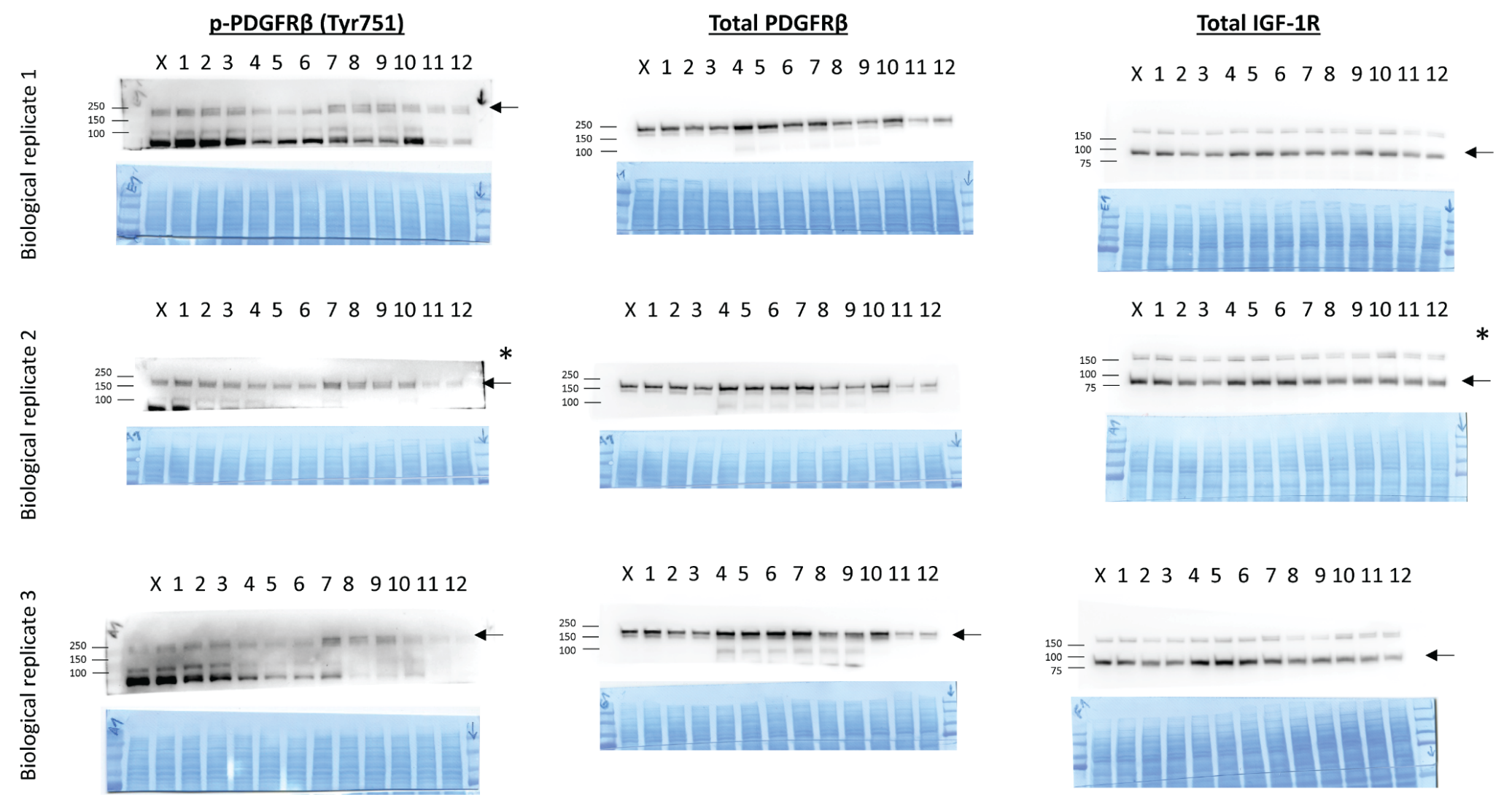


**Supplementary Data 5:** Uncropped Western blot images of p-AKT (Ser473), AKT and p-ERK (Ser202/204) immunodetection in SH-SY5Y cell line. GAPDH or α-tubulin served as loading control (see Supplementary Data 12 and 14) and Coomassie stained membranes are included to show loading for respective membranes. The experiments were repeated 3 times. * blots used in Figure 7A. 1: Ctrl; 2: Dp44mT; 3: DpC; 4: SUN; 5: SUN+Dp44mT; 6: SUN+DpC; 7: GEF; 8: GEF+Dp44mT; 9: GEF+DpC; 10: LAP; 11: LAP+Dp44mT; 12: LAP+DpC.


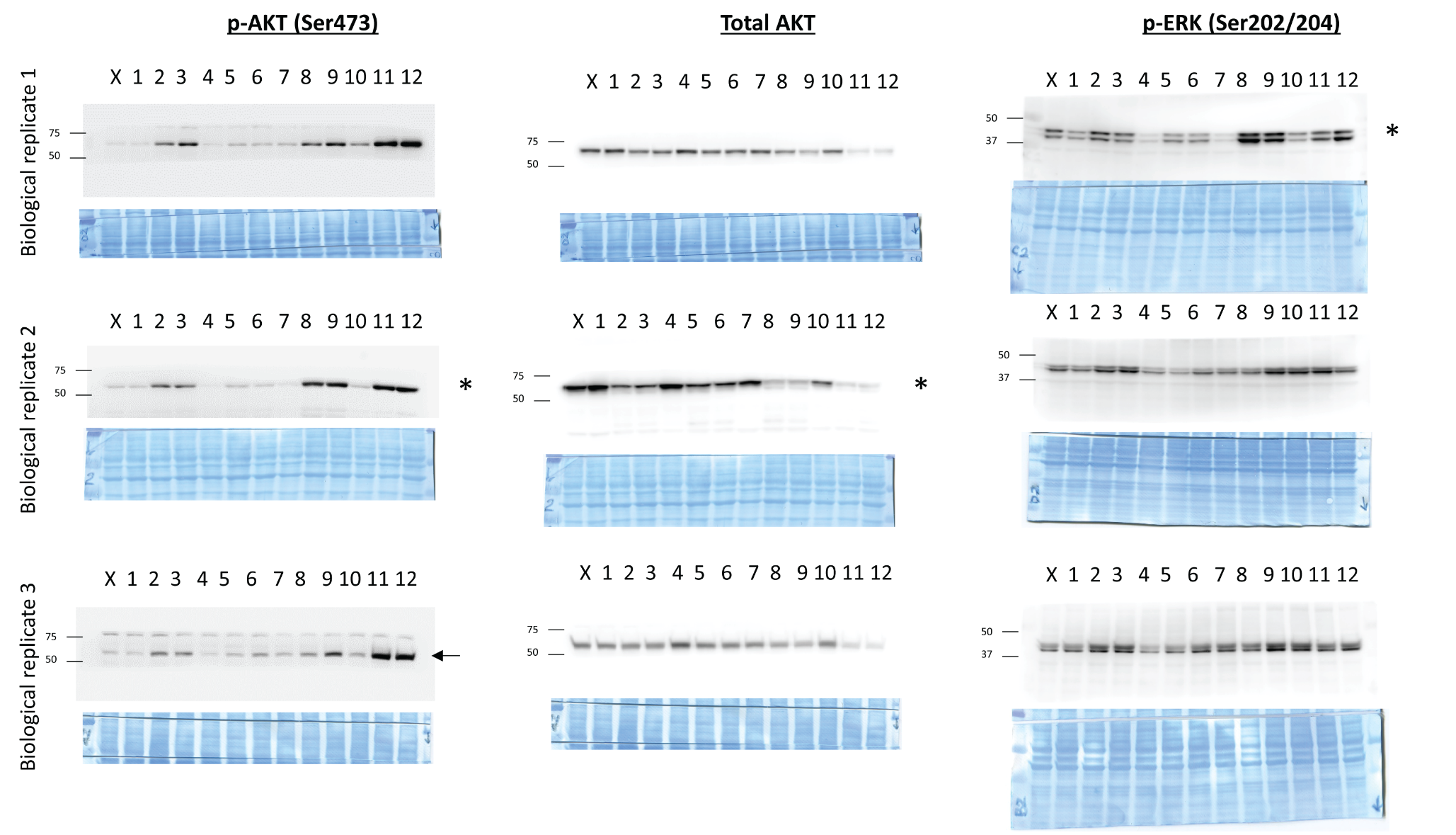


**Supplementary Data 6:** Uncropped Western blot images of p-MEK (Ser217/221), MEK and ERK immunodetection in SH-SY5Y cell line. GAPDH or α-tubulin served as loading control (see Supplementary Data 12 and 14) and Coomassie stained membranes are included to show loading for respective membranes. The experiments were repeated 3 times. * blots used in Figure 7A. 1: Ctrl; 2: Dp44mT; 3: DpC; 4: SUN; 5: SUN+Dp44mT; 6: SUN+DpC; 7: GEF; 8: GEF+Dp44mT; 9: GEF+DpC; 10: LAP; 11: LAP+Dp44mT; 12: LAP+DpC.


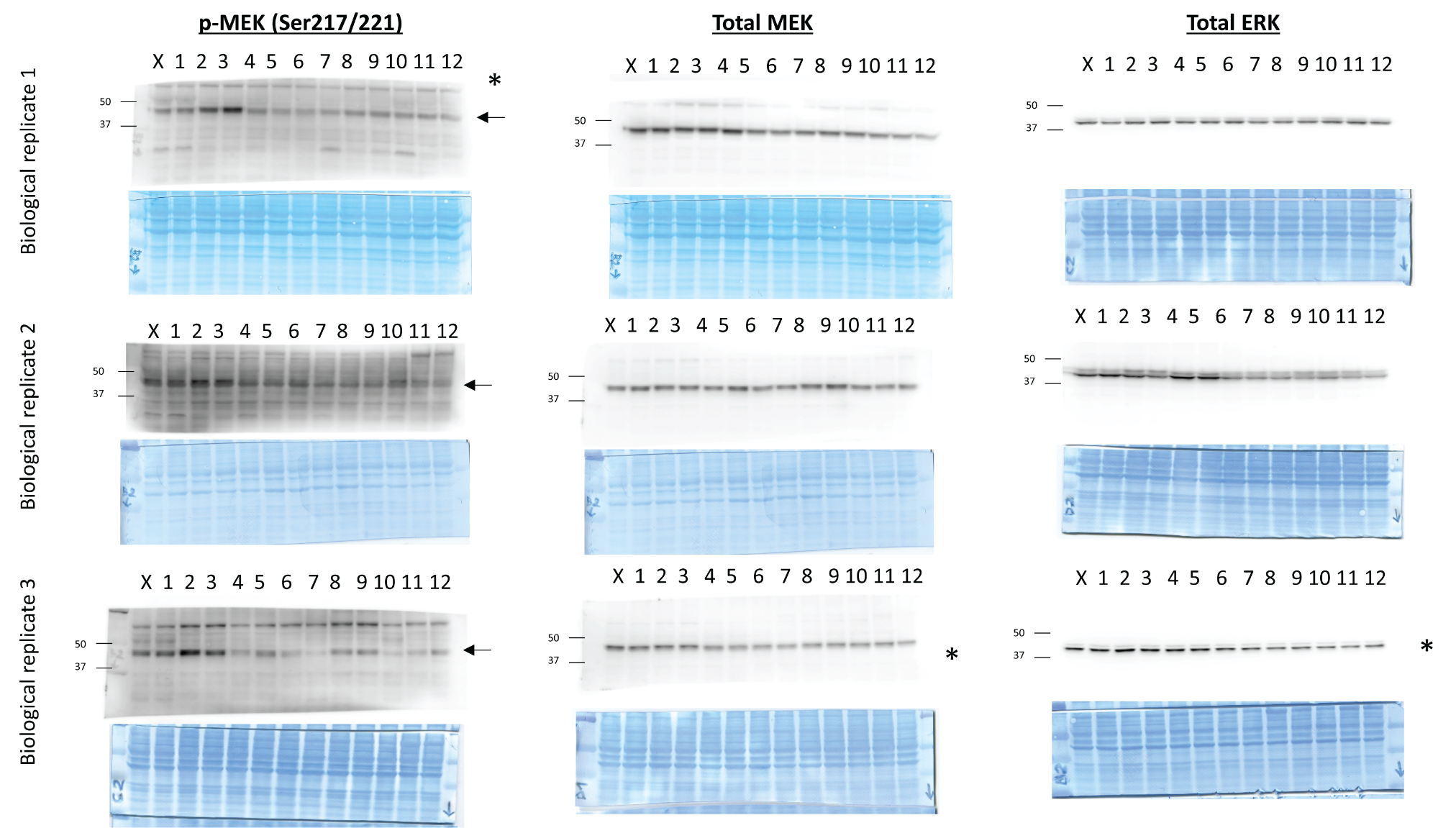


**Supplementary Data 7:** Uncropped Western blot images of p-AKT (Ser473), AKT and p-ERK (Ser202/204) immunodetection in SK-N-BE(2) cell line. GAPDH or α-tubulin served as loading control (see Supplementary Data 13 and 14) and Coomassie stained membranes are included to show loading for respective membranes. The experiments were repeated 3 times. * blots used in Figure 7B. 1: Ctrl; 2: Dp44mT; 3: DpC; 4: SUN; 5: SUN+Dp44mT; 6: SUN+DpC; 7: GEF; 8: GEF+Dp44mT; 9: GEF+DpC; 10: LAP; 11: LAP+Dp44mT; 12: LAP+DpC.


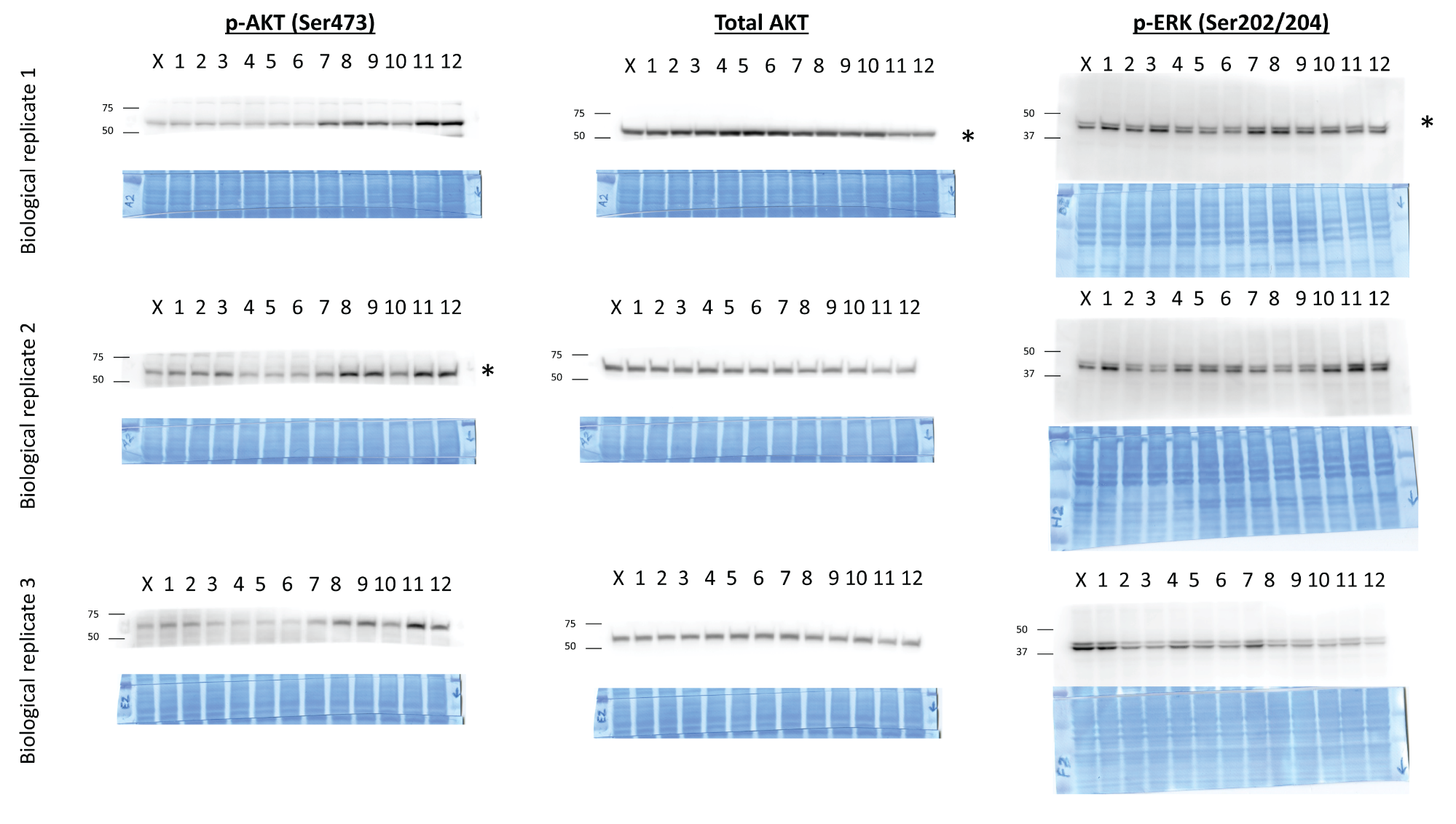


**Supplementary Data 8:** Uncropped Western blot images of p-MEK (Ser217/221), MEK and ERK immunodetection in SH-SY5Y cell line. GAPDH or α-tubulin served as loading control (see Supplementary Data 12 and 14) and Coomassie stained membranes are included to show loading for respective membranes. The experiments were repeated 3 times. * blots used in Figure 7A. 1: Ctrl; 2: Dp44mT; 3: DpC; 4: SUN; 5: SUN+Dp44mT; 6: SUN+DpC; 7: GEF; 8: GEF+Dp44mT; 9: GEF+DpC; 10: LAP; 11: LAP+Dp44mT; 12: LAP+DpC.


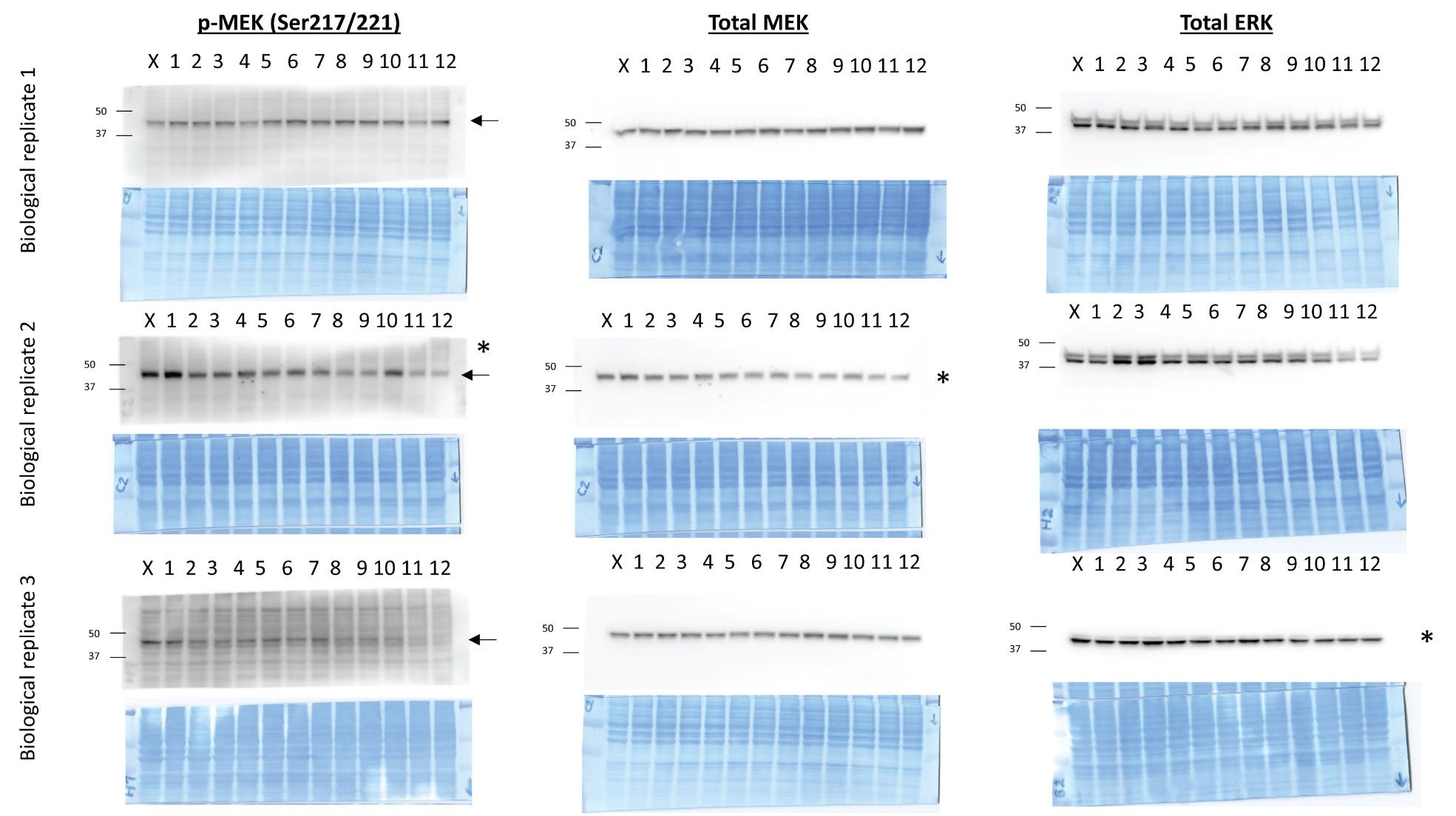


**Supplementary Data 9:** Uncropped Western blot images of cleaved caspase 3 in SH-SY5Y and SK-N-BE(2) cell lines. GAPDH or α-tubulin served as loading control (see Supplementary Data 12, 13 and 14) and Coomassie stained membranes are included to show loading for respective membranes. The experiments were repeated 3 times. * blots used in Figure 4. 1: Ctrl; 2: Dp44mT; 3: DpC; 4: SUN; 5: SUN+Dp44mT; 6: SUN+DpC; 7: GEF; 8: GEF+Dp44mT; 9: GEF+DpC; 10: LAP; 11: LAP+Dp44mT; 12: LAP+DpC.


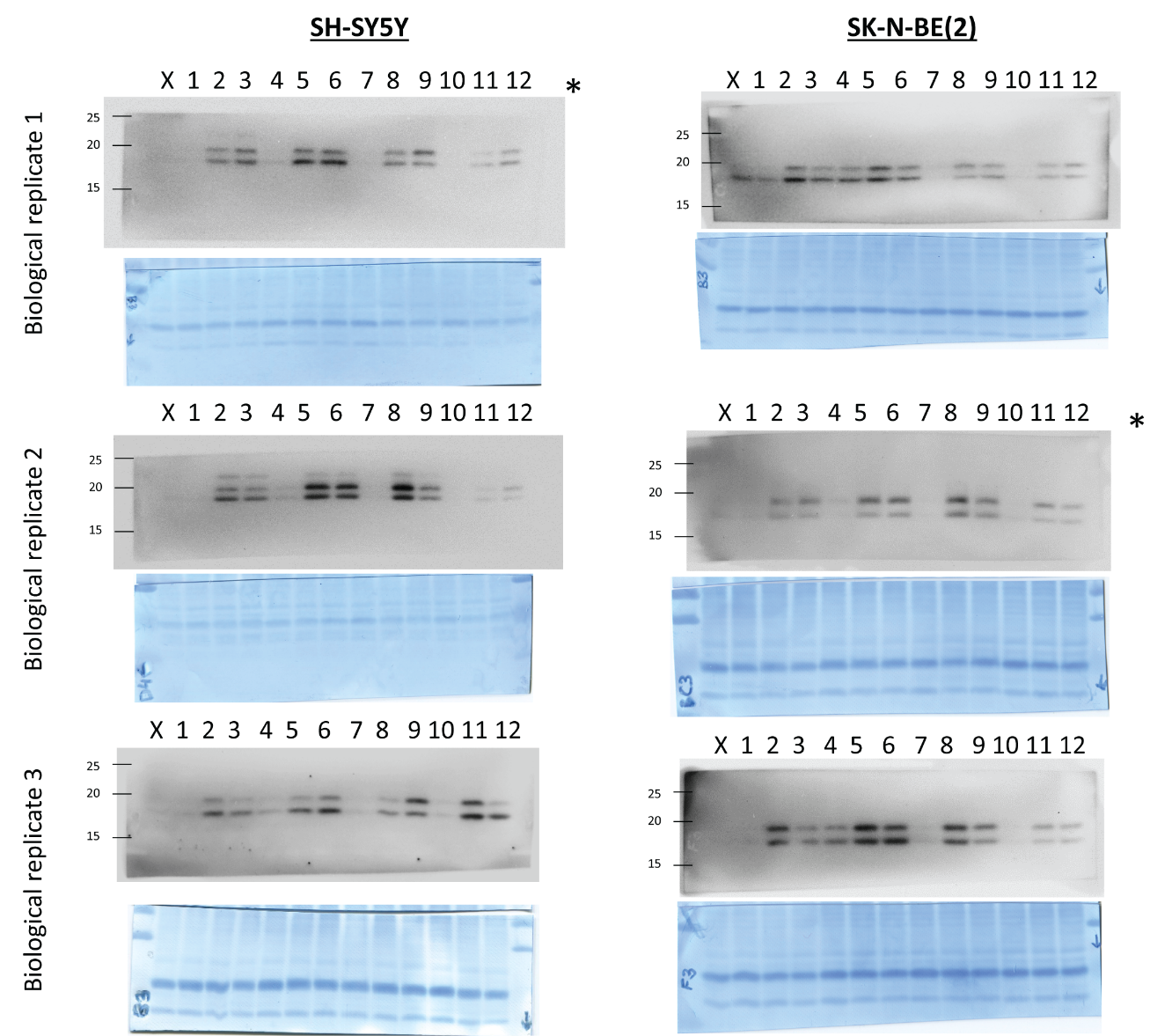


**Supplementary Data 10:** Uncropped Western blot images of p-NDRG1 (Thr346) in SH-SY5Y cell line. GAPDH or α-tubulin served as loading control (see Supplementary Data 12 and 14) and Coomassie stained membranes are included to show loading for respective membranes. The experiments were repeated 3 times. * blots used in Figure 8A. 1: Ctrl; 2: Dp44mT; 3: DpC; 4: SUN; 5: SUN+Dp44mT; 6: SUN+DpC; 7: GEF; 8: GEF+Dp44mT; 9: GEF+DpC; 10: LAP; 11: LAP+Dp44mT; 12: LAP+DpC.


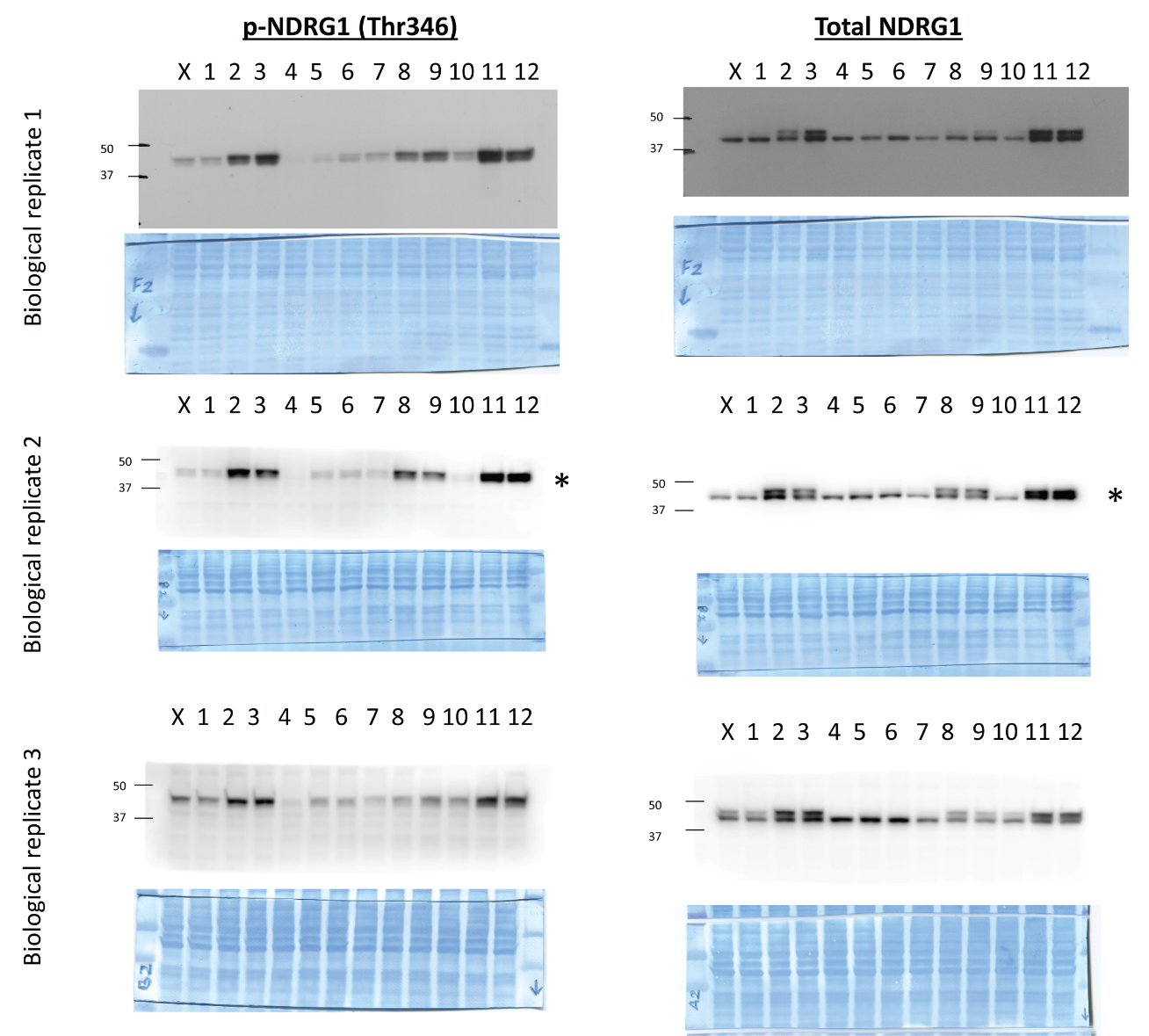


**Supplementary Data 11:** Uncropped Western blot images of p-NDRG1 (Thr346) in SK-N-BE(2) cell line. GAPDH or α-tubulin served as loading control (see Supplementary Data 13 and 14) and Coomassie stained membranes are included to show loading for respective membranes. The experiments were repeated 3 times. * blots used in Figure 8B. 1: Ctrl; 2: Dp44mT; 3: DpC; 4: SUN; 5: SUN+Dp44mT; 6: SUN+DpC; 7: GEF; 8: GEF+Dp44mT; 9: GEF+DpC; 10: LAP; 11: LAP+Dp44mT; 12: LAP+DpC.


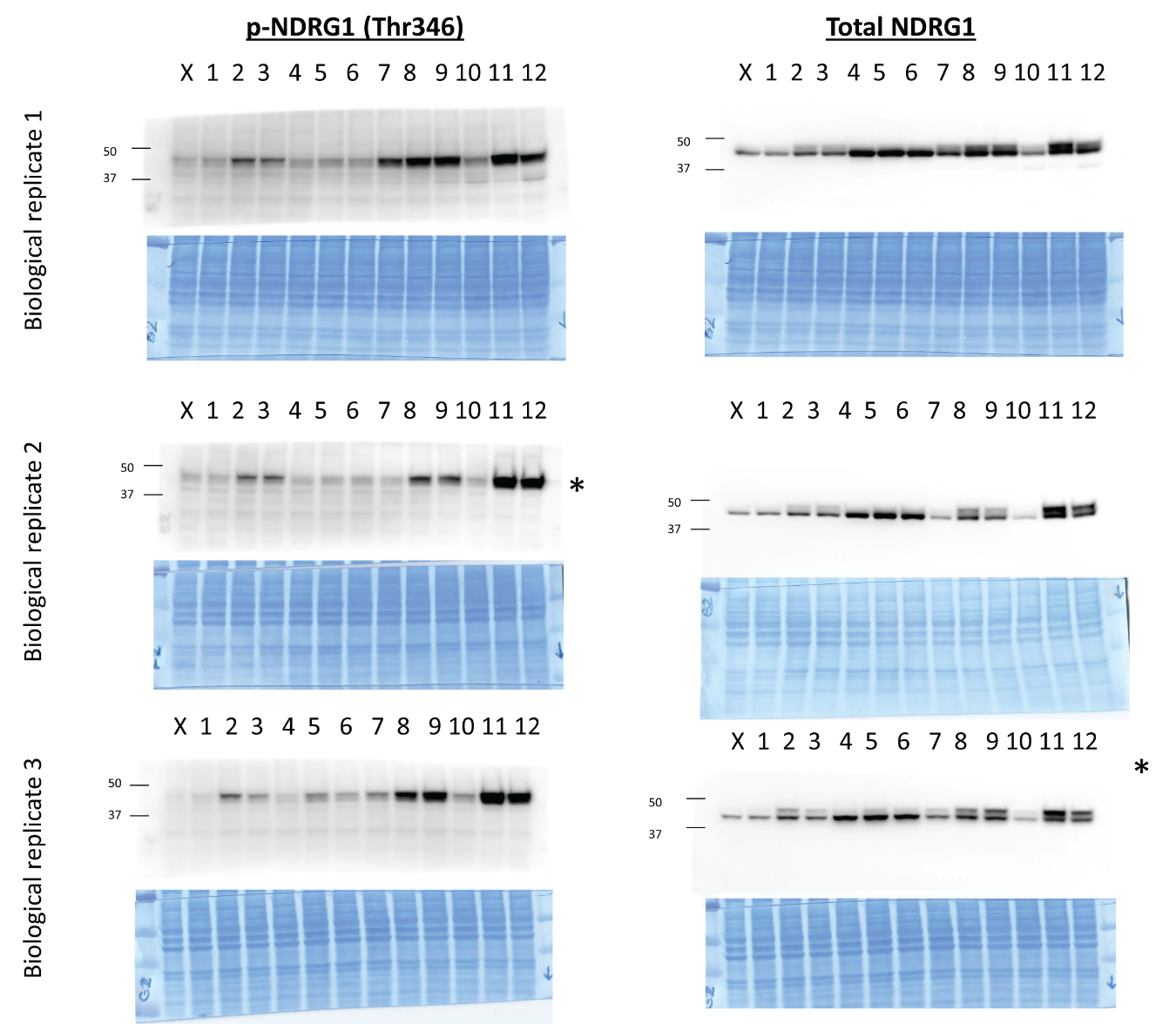


**Supplementary Data 12:** Uncropped Western blot images of GAPDH in SH-SY5Y cell line. 1: Ctrl; 2: Dp44mT; 3: DpC; 4: SUN; 5: SUN+Dp44mT; 6: SUN+DpC; 7: GEF; 8: GEF+Dp44mT; 9: GEF+DpC; 10: LAP; 11: LAP+Dp44mT; 12: LAP+DpC.


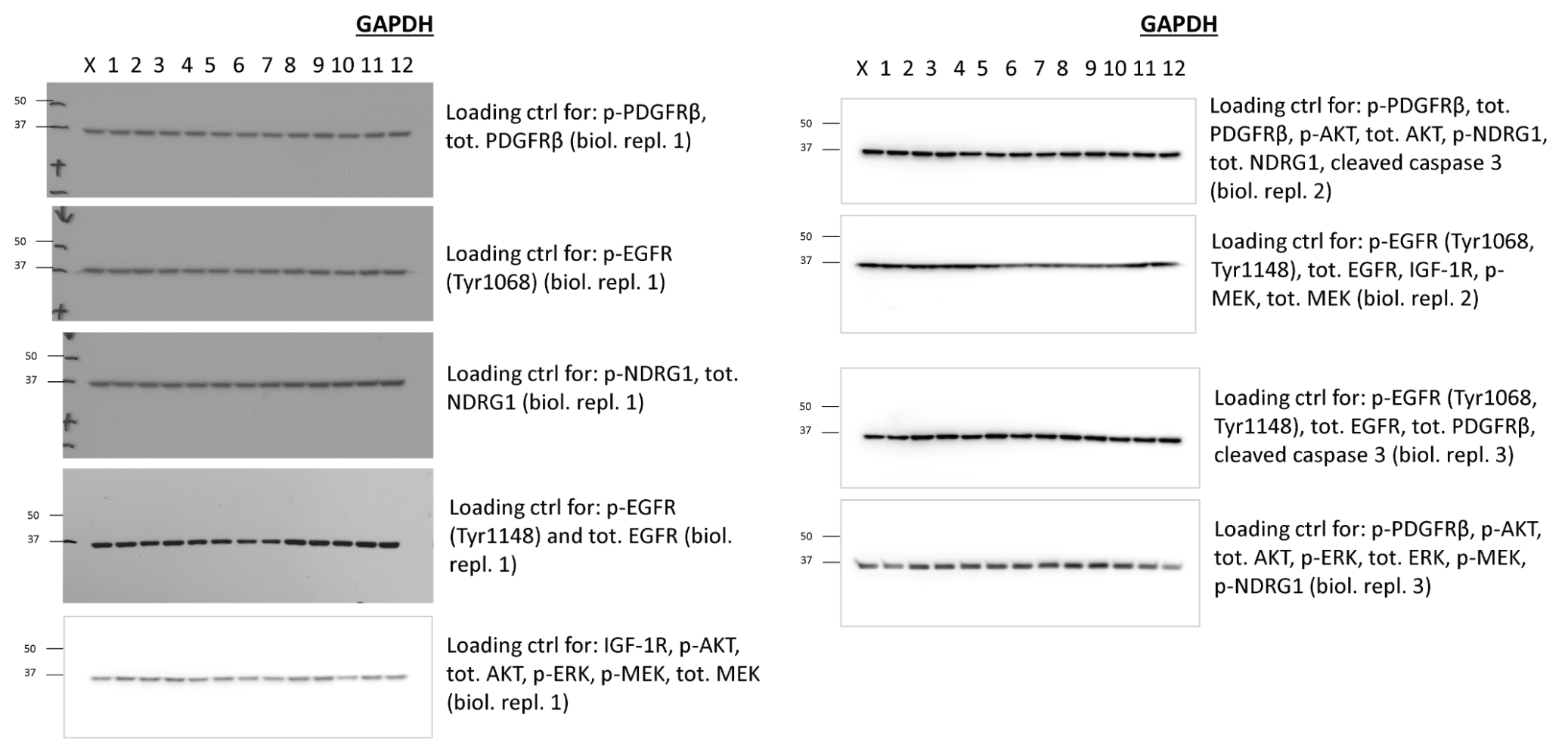


**Supplementary Data 13:** Uncropped Western blot images of GAPDH in SK-N-BE(2) cell line. 1: Ctrl; 2: Dp44mT; 3: DpC; 4: SUN; 5: SUN+Dp44mT; 6: SUN+DpC; 7: GEF; 8: GEF+Dp44mT; 9: GEF+DpC; 10: LAP; 11: LAP+Dp44mT; 12: LAP+DpC.


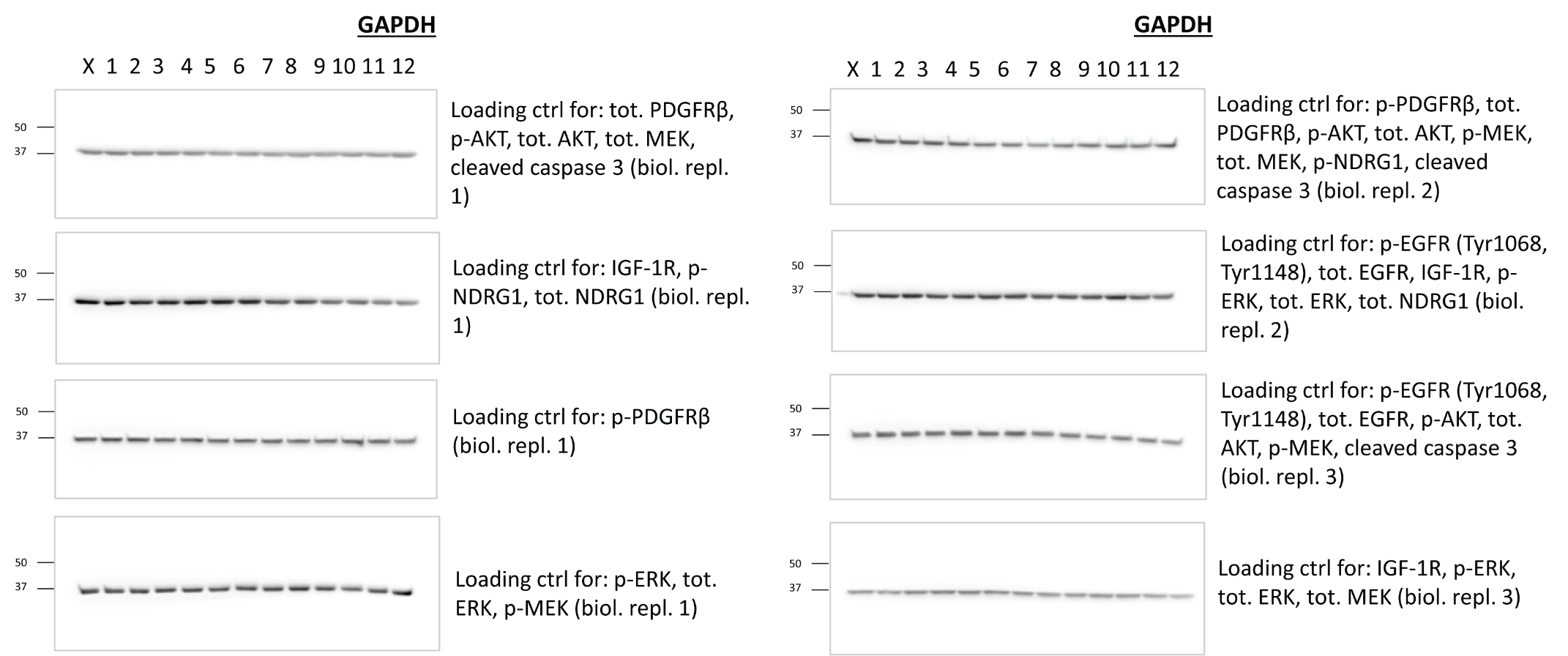


**Supplementary Data 14:** Uncropped Western blot images of α-tubulin inSH-SY5Y and SK-N-BE(2) cell lines. 1: Ctrl; 2: Dp44mT; 3: DpC; 4: SUN; 5: SUN+Dp44mT; 6: SUN+DpC; 7: GEF; 8: GEF+Dp44mT; 9: GEF+DpC; 10: LAP; 11: LAP+Dp44mT; 12: LAP+DpC.


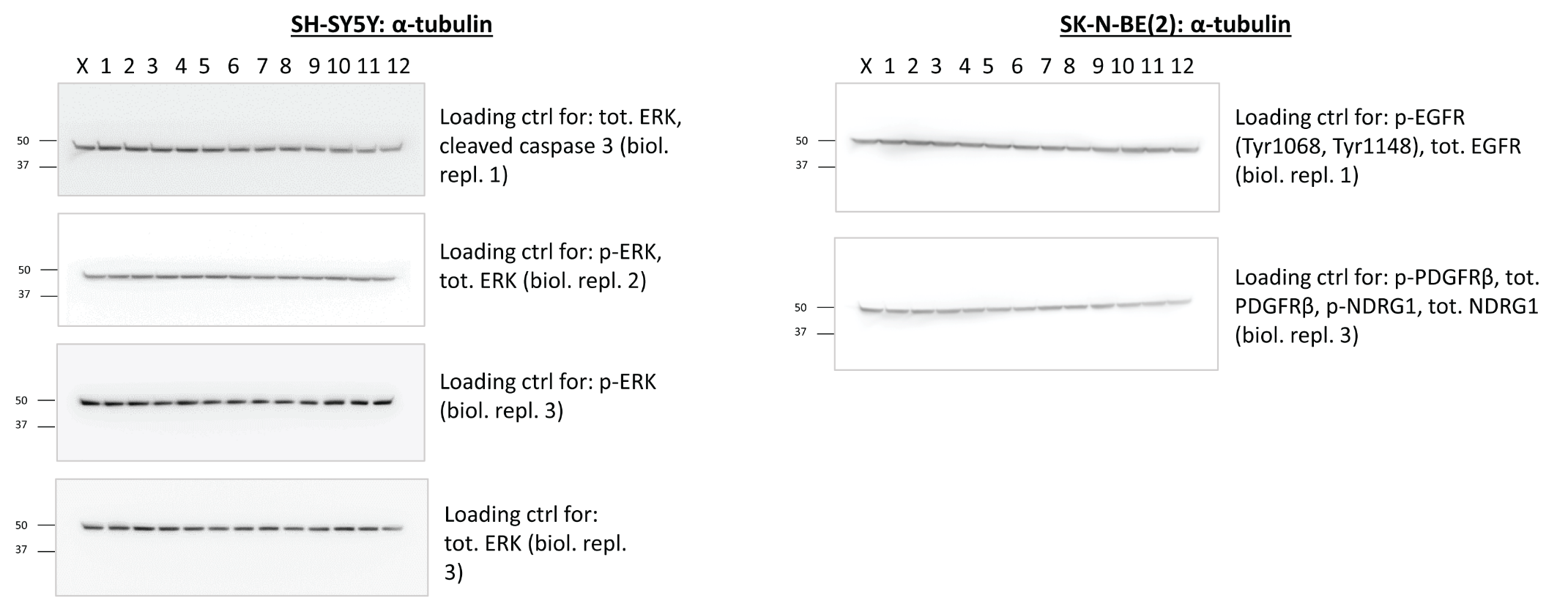

Supplement: Supplementary file 1 [file DataSheet1.docx]
